# Health policies and reforms in Mexico (2000-2024): segmentation and challenges for universal rights

**DOI:** 10.1590/0102-311XEN278225

**Published:** 2026-08-10

**Authors:** Eduarda Ferreira dos Anjos, Oliva López-Arellano, Elzo Pereira Pinto, Cristiani Vieira Machado

**Affiliations:** 1 Escola Nacional de Saúde Pública Sergio Arouca, Fundação Oswaldo Cruz, Fiocruz, Rio de Janeiro, Brasil.; 2 Instituto Gonçalo Moniz, Fundação Oswaldo Cruz, Fiocruz, Salvador, Brasil.; 3 Universidad Autónoma Metropolitana, Ciudade de México, México.

**Keywords:** Universal Access to Health Care Services, Health Services Coverage, Services Utilizations, Public-Private Partnerships, Latin America, Acceso Universal a los Servicios de Salud, Cobertura de los Servicios de Salud, Utilización de Servicios, Aparcería Público-Privada, Latin América

## Abstract

The Mexican health system is historically segmented, being based on social insurance for formal workers and public health actions for the general population. This study characterizes health reforms in Mexico from 2000 to 2024 considering the trajectory of health policies, coverage, and use of the health system. This research involved a literature review, documentary analysis, secondary data and interviews with key informants. We observed moments of reform under the influence of various groups and government political orientations. From 2003 to 2018, the implementation of a Popular Health Insurance with limited supply of services for informal workers and the poor increased coverage but reiterated segmentation and inequalities. From 2018 to 2024, center-left governments extinguished popular insurance and adopted reform strategies that centralized actions in the Federal Government and integrated efforts between institutions and public services. The results also showed the growing use of private clinics adjacent to pharmacies. In the period, the adopted reforms failed to overcome the legacy of segmentation by clientele, organizational fragmentation, and inequalities in health. Despite the announcement in April 2026 of reforms toward universal health some challenges can still be noticed, namely: insufficient public funding, limitations in infrastructure and the health workforce in the states, resistance from states and social security institutions, federative and political-party conflicts, expansion of private services under scarce regulation, and territorial inequalities and inequalities between social groups.

## Abstract (es)

El sistema de salud mexicano se caracteriza por su segmentación histórica, basada en el seguro social para los trabajadores formales y los programas de salud pública para la población en general. Este artículo caracteriza las reformas sanitarias en México entre el 2000 y el 2024, considerando la trayectoria de las políticas de salud, la cobertura y la utilización del sistema de salud. La investigación incluyó una revisión bibliográfica, un análisis documental y un análisis de datos secundarios, complementados con entrevistas a informantes clave. Se observaron diferentes momentos de reforma, influenciados por distintos grupos y orientaciones políticas de los gobiernos. Entre el 2003 y el 2018, la implementación de un Seguro Popular de Salud dirigido a trabajadores informales y pobres, con una oferta limitada de servicios, condujo a una mayor cobertura, pero reforzó la segmentación y las desigualdades. Entre el 2018 y el 2024, bajo gobiernos de centroizquierda, se extinguió el seguro popular y se adoptaron estrategias de reforma que implican la centralización de funciones en el Gobierno Federal y esfuerzos para integrar las instituciones y los servicios públicos. Los resultados también mostraron una expansión en el uso de consultorios privados adyacentes a las farmacias. Se concluye que, durante este período, las reformas adoptadas no lograron superar el legado de segmentación por clientelas, la fragmentación organizacional y las desigualdades en salud. A pesar del anuncio en abril del 2026 de reformas destinadas a universalizar la salud, persisten como desafíos la financiación pública insuficiente, las limitaciones en la infraestructura y en la fuerza de trabajo de salud en los estados, resistencias de los estados y de las instituciones de seguridad social, los conflictos federales y político-partidistas, la expansión de los servicios privados bajo una escasa regulación y las desigualdades territoriales y entre los grupos sociales.

## Introduction

Mexico is the second most populous country in Latin America, with more than 126 million inhabitants, and the region’s second-largest economy. Despite this, in 2020, approximately 43.9% of the population lived in poverty, 22.5% lacked access to quality food, and 28.2% did not have guaranteed access to health services. The country is vast and marked by diversity and inequalities across multiple dimensions, including territorial, socioeconomic, and ethnic-racial disparities. Mexico is a federation composed of 32 states and 2,475 municipalities, which poses challenges for the coordination of public policies. It is a presidential republic whose political history has been marked by a long period of a one-party state and, over the last two decades, by alternation between governments, reflected in the direction of health policies ^1^.

The Mexican health system has historically been characterized by segmented client groups, organizational fragmentation, and inequalities in service provision, coverage, and health outcomes ^2^. Key milestones in its organization include the creation of the Secretary of Health − later renamed Department of Health, equivalent to the Ministry of Health − and the Mexican Social Security Institute (IMSS, acronym in Spanish) in 1943. Over the years, other social security institutions were created to manage retirement benefits and pensions and to provide health services to formal workers: the Institute for Social Security and Services for Government Workers (ISSSTE, acronym in Spanish; 1960), Petróleos Mexicanos (PEMEX, acronym in Spanish; 1950), the Secretariat of the Navy (MARINA, acronym in Spanish; 1965) and the Secretariat of National Defense (SEDENA, acronym in Spanish; 1976) ^3^.

Despite their importance, until the 1980s social security institutions covered about 50% of the population, while millions of Mexicans without social security relied on public services with limited coverage and uneven distribution across states. Throughout the 1980s and 1990s, under governments led by the Institutional Revolutionary Party (PRI, acronym in Spanish), neoliberal reforms affected the economy, as well as social and health policies, promoting the decentralization of responsibilities and services to the states under inadequate institutional and financing conditions ^4^.

Beginning in the 2000s, with the election of governments of different political orientations, the Mexican healthcare system underwent reforms that sought to expand coverage for the population without social security, without breaking with the corporate structure of institutions responsible for social security and healthcare. In the context of neoliberal reforms, a Popular Health Insurance was implemented under the argument of achieving universal health coverage, in line with recommendations from international agencies for reforms in Latin America ^5^. As of 2018, a change of government led to the dismantling of this insurance and to institutional reforms guided by a different orientation ^6^ ^,^ ^7^.

This study characterizes health policies in Mexico between 2000 and 2024, a period marked by disputes over projects and health system reforms with different objectives. It seeks to analyze continuities and changes in the context and reform agendas over time, as well as to explore their effects on the institutional segmentation of health coverage and the use of different types of health services.

## Methodology

A case study of the Mexican health system was conducted. Mexico was selected due to its importance in Latin America, the pressures exerted by neoliberal agendas on health since the 1980s, and the dynamism of its health reforms in recent decades. The study period (2000-2024), comprises transitions of power between governments with different political orientations ^7^.

The analytical framework of the study is based on the literature on health policy analysis in Latin America, considering that health systems result from historical processes in which institutions influence actors’ strategies, as well as the trajectory and content of policies in each context. Such policies result from institutional norms, decision-making processes, government actions, and actions of interest groups ^8^. Therefore, the analysis of sectoral policies requires an understanding of rules, structures, and mobilization of resources and actors, while considering historical legacies and present-day decisions ^9^.

The analytical axes of the study were the historical-institutional trajectory of health policies and health system coverage and use across population groups.

Health policies during the period were analyzed in three phases: creation and implementation of Popular Health Insurance (2000-2018); creation and implementation of the Institute of Health for Welfare (INSABI, acronym in Spanish; 2019-2022); and its replacement by IMSS-Bienestar (2023-present). The characterization of health system coverage was conducted for the entire study period, considering national- and state-level data.

The research involved a bibliographic review of books and journals in the Virtual Health Library, Embase, PubMed, SciELO, Web of Science, and Scopus databases, as well as an analysis of official documents from 2000 to 2024, including legislation, reports, and resolutions.

Health system coverage and use were systematized based on publicly available official secondary data at both the national and state levels. Data on population coverage by type of institutional affiliation were obtained from the Census conducted by the National Institute of Statistics and Geography (INEGI, acronym in Spanish) for the years 2000, 2005, 2010, and 2020. Data on healthcare use were extracted from the question “health services/institutions usually sought in case of health needs” included in the *National Health and Nutrition Survey* (ENSANUT, acronym in Spanish; *Encuesta Nacional de Salud y Nutrición*), a sample survey, for the years 2000, 2012, 2020, and 2023. Descriptive analyses were performed, with percentage calculations and preparation of tables using Microsoft Excel (https://products.office.com/).

Over a two-week period in April 2024, fieldwork was conducted in Mexico, with direct observation in public and private healthcare facilities, participation in technical meetings related to the ongoing reform, and interaction with researchers, health professionals, and municipal managers in Mexico City and other regions of the country. Additionally, four semi-structured interviews were conducted with key informants − three in person during fieldwork and one online − guided by a script addressing the contexts and reform processes. Interviewees were selected based on their knowledge of and involvement in health system reforms during the study period and included two researchers on the topic who had worked as managers in Mexico City; one researcher from the National Institute of Public Health; and one representative of an international organization that had followed the recent reform. The study was approved by the Research Ethics Committee of the Oswaldo Cruz Foundation (Fiocruz, CAE n. 18083719.9.0000.5240).

## Results

### Popular Health Insurance: pursuing universal health coverage (2000-2018)

Mexico was a special case in Latin America in adopting Popular Health Insurance, aimed at covering poor populations and informal workers to provide access to a limited package of health services and medications. Without breaking with the corporate arrangement by which formal workers received health coverage via social security institutions, the implementation of Popular Health Insurance sought to achieve universal coverage and to prevent household impoverishment resulting from high out-of-pocket health expenditures.

The Popular Health Insurance experience emerged in the context of the rise to power of the right-wing National Action Party (PAN, acronym in Spanish). Vicente Fox was elected President for the 2000-2006 term after defeating the candidate of PRI, which had occupied the Federal Government for 71 years ^4^. His administration was marked by a liberal economic agenda, labor market flexibility, outsourcing of social security services, and health reforms ^10^.

In the health sector, Júlio Frenk was appointed Minister of Health. He had previously served as director of INSP (1987-1992), consultant to the World Bank (1995-1996), and executive vice president of the Mexican Health Foundation (Funsalud, acronym in Spanish) (1995-1998) ^10^ ^,^ ^11^. Reforms implemented under his leadership sought to establish a Social Protection System in Health (SPSS, acronym in Spanish). Key strategies included legal changes ^12^ aimed at ensuring financial sustainability, an emphasis on efficiency, the creation of Popular Health Insurance, and a new wave of administrative decentralization, under which the states assumed responsibility for managing Popular Health Insurance, including receiving federal funds, providing financial counterpart contributions, enrolling families, and directly managing or contracting health services. Economic and administrative policies maintained wage restraint and high levels of labor informality, undermining the corporate health system ^10^ ^,^ ^13^.

To regulate the implementation of the General Health Law (LGS, acronym in Spanish), the Federal Commission for Protection Against Health Risks (Cofepris, acronym in Spanish) was created, with technical, operational, and administrative autonomy ^12^. During this period, the number of regulatory agencies in Mexico increased; however, this did not translate into better resource management, financial efficiency, or a stronger role of the State ^13^. The 2001 LGS introduced the possibility of multi-year contracts, promoting public-private partnerships via legal agreements that could extend beyond a single fiscal year and allowed the State to purchase or lease private goods and services ^10^ ^,^ ^14^. This contributed to reduced investment in infrastructure and increased private-sector participation via service outsourcing ^1^. Funsalud, a nonprofit business association, proposed the creation of a private fund for public health programs and influenced the establishment of the SPSS, whose operational arm was Popular Health Insurance ^7^.

Popular Health Insurance underwent three phases: launch (2000-2006), consolidation (2006-2012), and decline (2012-2018) ^1^. Its implementation, which depended on voluntary adherence by federative entities, began in five states in 2001 and reached all 32 states in 2004. Mexico City was the last entity to join due to the resistance from then head of government Andrés Manuel López Obrador, who at the time was affiliated with the opposition Democratic Revolution Party (PRD) ^15^.

The National Commission for Social Protection in Health and the State Regimes for Social Protection in Health, at the federal and state levels respectively, were responsible for implementing the system, ensuring financing and service provision by using multi-year contracts ^4^ ^,^ ^16^.

Popular Health Insurance financing was tripartite, consisting of per capita federal and state contributions, as well as contributions from insured families/individuals proportional to income, with exemptions granted upon proof of inability to pay. The number of enrolled families determined the states’ per capita allocation ^17^. Regarding service provision, the covered package of essential services was defined by the Medical Benefits Catalog, which consisted of 78 health interventions (2002-2003) and 191 medications and vaccines. This package was later expanded and, by 2019, the Universal Catalog of Health Services (CAUSES, acronym in Spanish) covered 294 interventions and 670 medications, vaccines, and supplies ^18^. Oversight of delivery service and implementation of CAUSES was the responsibility of the states, which were also charged with coordinating and contracting healthcare providers to deliver care to insured individuals ^19^. Despite this expansion, such services covered remained inferior to those offered by IMSS and ISSSTE, with inequalities across providers.

The consolidation period of Popular Health Insurance ^1^ (2006-2012) corresponded to the administration of Felipe Calderón, also from PAN, whose election was marked by a narrow margin of victory over Andrés Manuel López Obrador, triggering political polarization and disputes over the results ^5^. Reforms adopted during this period sought to ensure financial sustainability, optimization of resources, and management capacity ^10^. Commitments were made to reduce maternal and under-five mortality, as well as to combat diseases such as HIV/AIDS, malaria, and tuberculosis. The Ministry of Health established indicators to monitor the performance of state-level services and adopted a financing model partially based on performance evaluation and outcomes ^20^.

Popular Health Insurance expanded enrollment, the number of interventions covered by CAUSES, and its budget until 2010. It relied on outpatient services provided by state and contracted private health departments, as well as on specialized services and hospitals that were predominantly private ^2^. In its early years, the program succeeded in prioritizing lower-income families and reducing out-of-pocket spending on medications ^6^. However, investments in IMSS and ISSSTE remained higher, with government per capita spending in the social security system exceeding that allocated to Popular Health Insurance ^20^.

The reform measures maintained a liberal orientation and sought to improve the system’s management capacity, financial sustainability, and resource utilization. In 2012, the Commission for Social Protection in Health announced that Mexico had achieved universal health coverage, with approximately 52 million people enrolled in Popular Health Insurance ^2^ ^,^ ^3^. However, the reported achievements were controversial, as some analyses indicated that 25% of the population still lacked any form of health insurance ^2^.

The decline phase of Popular Health Insurance ^7^ coincided with the return of PRI to power under President Enrique Peña Nieto (2012-2018). His administration promised modernization, transparency, and the reduction of violence. In this context, structural reforms opened greater space for private capital in the energy, communications, and education sectors ^4^.

In the health sector, physician Mercedes López was appointed Minister of Health. She had previously served as executive president of Funsalud and was committed to building a Universal National Health System (Sinasu). The Health Sector Program (2013-2018) identified three major problems: a curative rather than preventive healthcare model, incomplete implementation of multidisciplinary policies, and unequal governance and financial arrangements ^10^.

In response, the Comprehensive Healthcare Model was designed in 2015 with the goal of standardizing services and practices via person-centered care, optimization of resources and infrastructure, and strengthening of Local Health Systems and Integrated Health Service Networks ^20^. Investments were directed toward strengthening cost-effective primary care with projects focused on family medicine and primary healthcare (PHC). This organizational model sought to implement care networks and guidelines for patient follow-up, particularly for people with chronic diseases, and to expand access to PHC, despite the persistence of several other unresolved barriers ^10^.

Insufficient infrastructure for service provision within Sinasu led to the approval of the Public-Private Partnership Law, which enabled the construction of hospitals operated by IMSS while granting advantages to the private sector ^21^. Other measures included joint procurement processes among the health subsystems for the purchase of medications, aimed at improving cost-effectiveness and transparency ^10^.

Reforms related to IMSS sought to strengthen its financial sustainability as a public-commercial institution ^10^. By 2016, the number of individuals enrolled in Popular Health Insurance had reportedly reached approximately 52 million ^6^; however, its structural problems had become increasingly evident. The country’s federal arrangement produced 32 distinct health subsystems, marked by disparities across states in per capita funding, management capacity, human resources, medical and hospital infrastructure, and availability and quality of services ^2^. High levels of out-of-pocket spending persisted, without the effective achievement of universal health coverage. Thus, by the end of the Peña Nieto administration, a period marked by substantial expansion of the private sector financed with public resources ^3^, the failure of reforms to universalize access to health services, ensure financial protection, and overcome inequalities had become clear ^22^. Changes within IMSS and the management of Popular Health Insurance ultimately rendered Sinasu unfeasible, particularly due to infrastructure and financing constraints.

### From INSABI to IMSS-Bienestar: in search of a Universal Public System (2018-2024)

The National Regeneration Movement Party (MORENA), founded in 2011 by Andrés Manuel López Obrador, was the first left-wing party to assume the Federal Government after a long period of center-right and right-wing rule. During his campaign, Obrador proposed a “fourth transformation” centered on eradicating corruption, strengthening the state, and building a fair health system, including the dismantling of Popular Health Insurance, which he described as “*ni es seguro, ni es popular*” (neither insurance nor popular) ^20^. Physician Jorge Alcocer Varela was appointed Minister of Health and tasked with reforming the National Health System (SNS, acronym in Spanish) ^4^. The group associated with the Latin American Social Medicine tradition and the struggle for the right to health gained greater influence, although different positions regarding reform strategies emerged throughout the administration.

Popular Health Insurance ceased operations in 2019 and was replaced by INSABI, which was joined by 23 states ^23^ and remained in operation from 2019 to 2022, a period that largely overlapped with the COVID-19 pandemic ^24^. INSABI was a decentralized public agency whose primary objective was to guarantee the free provision of healthcare services to people without social security coverage. It was also responsible for supplying medications and health inputs and, together with the Ministry of Health, promoting integration and coordination within SNS ^23^. The guiding principles of this reform were universality (provision of necessary services to people without social security coverage), free access (delivery of public services without user fees), and anti-corruption (centralized control over procurement and service provision, with reduced private sector participation) ^20^.

Regarding financing, unlike the funding model of Popular Health Insurance − which was theoretically shared among the Federal Government, state governments, and enrolled individuals, but in practice was financed via approximately 80% federal and 20% state resources − INSABI did not provide for a separation of functions and operated in coordination with the state health secretariats. Resources from the Health for Well-Being Fund (Fonsabi, acronym in Spanish), financed via tax revenues, were designed to be transferred directly to providers (hospitals and medical units), with the aim of shifting financing from demand-side mechanisms toward the provision of public services ^20^.

The restructuring of the system dismantled previously autonomous bodies and transferred responsibilities to the Department of Health ^21^, generating tensions regarding the role of regulatory agencies. There were also differing positions among government supporters concerning the creation of a new institution, as opposed to expanding coverage by means of the existing social security institutions. Discrepancies were observed in the data regarding the number of affiliates and service coverage. The National Council for the Evaluation of Social Development Policy (Coneval, acronym in Spanish) identified an increase in the number of individuals reporting lack of access to health services, which may be associated with the dismantling of Popular Health Insurance and the absence of mandatory enrollment in INSABI. The lack of operational guidelines, the absence of its own service network, and uncertainties arising from both the institutional changes and the public health emergency were decisive factors in INSABI’s termination ^15^ ^,^ ^20^.

The response to the COVID-19 pandemic was hindered by institutional fragmentation and difficulties in Federal Coordination. Strategies focused on expanding hospital bed capacity, directly hiring personnel, and establishing public-private partnerships ^24^. Previous experience with the 2009 H1N1 outbreak proved relevant for planning and responding to this public health crisis ^23^ ^,^ ^24^.

The government hired 50,000 physicians and nurses, provided training, and supplied healthcare workers with tablets and mobile phones. Both the public and private sectors produced personal protective equipment, medical devices, masks, and other supplies. Although the Federal Government was slow to acknowledge the severity of the pandemic, mitigation measures were eventually adopted, including sector-based quarantine policies and the use of an epidemiological traffic light system ^23^ ^,^ ^24^.

Some of the major challenges in responding to the pandemic included insufficient diagnostic testing, limited mask use, and difficulties coordinating interventions, as well as the high prevalence of obesity, diabetes, and hypertension, which contributed to elevated COVID-19 lethality rates. Furthermore, influenza and pneumonia ranked among the leading causes of death during the period, and excess overall mortality was high, suggesting underreporting of COVID-19 cases ^24^.

Austerity measures implemented by the previous administration, together with longstanding problems in the medication procurement system, led to supply chain disruptions, public dissatisfaction, and protests ^7^ ^,^ ^20^. Limited public understanding of the INSABI reform and the constraints on its implementation, combined with preexisting problems such as underfunding, led to another reform process. INSABI was dismantled, and its resources, rights, and obligations were transferred to a new decentralized public agency, IMSS-Bienestar ^23^.

IMSS-Bienestar launched a pilot project in four states in which no change in government was expected in 2024, with the aim of ensuring the program’s stability and consolidation. The subsystem seeks to provide services to approximately 53.2 million Mexicans without social security coverage across the 23 Federative Entities that joined the initiative ^4^. The strategy involves a degree of recentralization of management and registration of citizens to inform them of their right to healthcare. Nine states governed by parties opposed to the Federal Government chose to retain the management and provision of services via their state health secretariats, with varying levels of capacity ^1^. Partial state adherence threatens the solidity of IMSS-Bienestar, reinforces stratification in coverage and access, and undermines the right to health.

The reform aims to ensure effective, timely, and high-quality access to healthcare services, including the right to comprehensive medical and hospital care, medications, and other health supplies free of charge. It is operationalized via the Health Care Model for Well-Being, which applies to all institutions within the system, with a focus on PHC. The model includes a surveillance component and may involve collaboration agreements with IMSS for the use of healthcare resources and equipment, promoting the sharing of infrastructure and greater integration between state public services and IMSS facilities ^25^. The Mexican health system also continues to rely on the contracting of private services, particularly for specialized procedures and diagnostic services, a practice that has persisted since the reforms in the 1990s ^10^.

The Governing and General Administration Board is responsible for managing IMSS-Bienestar and establishing its priorities, policies, organizational foundations, and constituent areas. Financing is tax-based and does not require enrollment contributions, with the aim of reducing inequalities in access generated under Popular Health Insurance. In addition to federal funding sources, state governments transfer funds directly to IMSS-Bienestar, thereby recentralizing resources in an effort to prevent mismanagement and corruption. Regarding structure, the National Agreement for the Federalization of the Health System for Well-Being established ^26^ that the national and state health secretariats would transfer their healthcare facilities, budgets, and personnel to IMSS-Bienestar, along with the incorporation of five regional hospitals and one specialized center.

The subsystem is organized into three levels of care, articulated via integrated health service networks. PHC constitutes the first level and includes one physician for every 3,000 individuals, in addition to nursing care, emergency services, family planning, dentistry, nutrition, social work, psychology, rehabilitation, and diagnostic support services. Second-level general hospitals provide care in general surgery, internal medicine, anesthesiology, pediatrics, gynecology, and obstetrics. The third level consists of specialized hospitals operating by means of referral and counter-referral mechanisms ^27^.

The ongoing changes have been maintained under the current administration of Claudia Sheinbaum, the first woman to serve as President of Mexico (2024-2030), elected by MORENA with a wide margin of votes. These reforms are aligned with the government’s development and health plans, whose objectives include consolidating a development model grounded in well-being, social justice, and sustainability. The appointed Minister of Health was David Kershenobich, a physician trained at National Autonomous University of Mexico (UNAM), who announced that the administration’s main objective would be to guarantee the right to health and well-being while aligning commitments with the 2030 Agenda and measurable targets ^27^. Characteristics of health reforms in Mexico during the period, highlighting elements of the context, political process, and adopted strategies, are summarized in Box 1.

**Box 1:** National context and health policies in Mexico, from 2000 to 2024.

MOMENTS	CONTEXT	HEALTH POLITICAL PROCESS	AGENDA - STRATEGIES
Popular Health Insurance 2000-2006 Launch	Vicente Fox Quesada Mandate - National Action Party (PAN, acronym in Spanish; Dec/01/2000 - Nov/30/2006) Political-Institutional Scenario: - Democratic transition - Macroeconomic stability - Strong influence of the World Bank on the reform agenda - High popularity Economic agenda: - Partial economic tax reform - Privatization of the energy sector and opening of private contracts in Petroles Mexicanos (PEMEX, acronym in Spanish) - Investment in assistance programs for the low-income population Health financing: In 2000: Total Health Expenditure % of GDP: 4.2% Public Health Expenditure % of GDP: 1.9% Private Expenditure % of Total Health Expenditure: 54.8% Direct Disbursement % Total Health Expenditure: 52.2%	Júlio Frenk - Minister of Health (Dec/01/2000 - Nov/30/2006) - Leadership of the “New Public Health”, with a trajectory at Funsalud, the National Institute of Public Health (INSP, acronym in Spanish) and acting in the reform of Colombia - Broad influence on health reforms - Influence of international agendas on reforms, with emphasis on achieving “universal health coverage” - Gradual adhesion of states, influenced by political-party conflicts	Managerial reforms, focus on expanding coverage for the poor population, and decentralization 2001: Publication of the first Institutional Program of the Mexican Institute of Social Security 2001-2006 (PIIMSS, acronym in Spanish) 2001: Creation of the Federal Commission for Protection against Health Risks (Cofepris), an important institution in the decentralization of the health system 2003: Implementation of Popular Health Insurance in favor of achieving Universal Health Coverage in the country 2004: Promulgation of the General Law of Social Development 2005: Creation of the National Council for the Evaluation of Social Policy (Coneval, acronym in Spanish), which played an important role in the acceptance and consolidation of Popular Insurance 2006: Integrative Model of Primary Health Care (MIDAS, acronym in Spanish) 2006: Federal Budget and Fiscal Responsibility Act (LFPRH, acronym in Spanish)
Popular Health Insurance 2006-2012 Consolidation	Felipe Calderón Hinojosa Mandate - PAN (Dec/01/2006 - Nov/30/2012) - Tax reform to increase revenue - Attempt at reform with flexibility of labor laws - Maintenance of private contracts at PEMEX - International economic crisis (2008-2009) Health financing: In 2006: Total Health Expenditure % of GDP: 5.4% Public Health Expenditure % of GDP: 2.3% Private Expenditure % of Total Health Expenditure: 57.2% Direct Disbursement % Total Health Expenditure: 53.8%	José Ángel Villalobos - Minister of Health (Dec/01/2006 - Sep/09/2011) - Federal deputy and president of the Health Commission in the Chamber of Deputies (2000-2003) - Affiliated to PAN - Defense of administrative efficiency Salomão Woldenberg - Minister of Health (Sep/09/2011 - Nov/30/2012) - Coordinated Popular Health Insurance (2009-2011)	Continuity of previous reforms, incentives for the participation of private services 2007: IMSS Institutional Program 2007-2012, aimed at modernization and separation of functions 2008: Creation of the Coordinating Commission for the Negotiation of Prices of Medicines and Other Health Inputs 2007: North America’s Plan for Avian and Pandemic Influenza (NAPAPI) 2009-2010: Response to the H1N1 health emergency 2012: Enactment of the Public-Private Association (APP, acronym in Spanish) Law; boosted private contracting to offer services
Popular Health Insurance 2012-2018 Decline	Enrique Peña Nieto - Institutional Revolutionary Party (PRI, acronym in Spanish; Dec/01/2012 - Nov/30/2018) - Energy and tax reform allowing private capital to enter Pemex; tax on sugary drinks - Completion of the labor reform initiated in the previous government - High violence rates - Reports of corruption Health financing: In 2012: Total Health Expenditure % of GDP: 5.4% Public Health Expenditure % of GDP: 2.9% Private Expenditure % of Total Health Expenditure: 46.9% Direct Disbursement % Total Health Expenditure: 40.6%	Mercedes Juan López - Minister of Health (Dec/01/2012 - Feb/08/2016) - First woman in office - Link with PRI - Held a management position at Funsalud José Narro Robles - Minister of Health (Feb/08/2016-Nov/30/2018) - Link with PRI	Continuity of the Popular Insurance model, emphasis on the achievement of universal health coverage and the care model 2012: The Commission on Social Protection in Health (CPSS, acronym in Spanish) announces the scope of Universal Health Coverage 2015: Comprehensive Health Care Model (MAIS, acronym in Spanish)
INSABI 2019-2022	Andrés Manuel López Obrador − National Regeneration Movement Party (MORENA, acronym in Spanish; (Dec/01/2018 - Sep/30/2024) - Set of reforms termed “Fourth Transformation” - Reforms to increase state regulation in the energy and PEMEX sectors, returning the status of public companies - Expansion of pension programs for older adults and vulnerable populations - Creation of new universities in the country - Late recognition of the importance of the COVID-19 pandemic - High national popularity Health financing: In 2019: Total Health Expenditure % of GDP: 5.3% Public Health Expenditure % of GDP: 2.6% Private Expenditure % of Total Health Expenditure: 50.8% Direct Disbursement % Total Health Expenditure: 42.3%	Jorge Alcocer Varela - Minister of Health (Dec/01/2018 - Sep/30/2024) - Strong influence of the Latin American social medicine group led by Asa Cristina Laurell, professor at UAM-X. Difficulties in articulating the new Institute with existing institutions and services.	Extinction of the Popular Health Insurance and creation of a new National Institute to universalize coverage 2018: Extinction of PI and creation of INSABI in favor of a Universal Health System 2019: Onset of the COVID-19 pandemic with high lethality rates and mortality in the country 2019: Changes in the purchase of medications 2022: Creation of legal bases of the IMSS-Bienestar and the Health Care Model for Bienestar (MAS-Bienestar)
IMSS-Bienestar 2023 - current	Andrés Manuel López Obrador - MORENA (Dec/01/2018 - Sep/30/2024) Cláudia Sheinbaum - MORENA (Oct/01/2024-current) - First woman president - High popularity - Had as a campaign promise the maintenance of reforms initiated by her predecessor, terming the measures “Second Part of the Fourth Transformation” Health financing: In 2022: (last year available) Total Health Expenditure % of GDP: 5.7% Public Health Expenditure % of GDP: 3.0% Private Expenditure % of Total Health Expenditure: 48.1% Direct Disbursement % Total Health Expenditure: 39.1%	Jorge Alcocer Varela - Minister of Health (Dec/01/2018 - Sep/30/2024) David Kershenobich - Minister of Health (Oct/01/2024-current) - Doctor from UNAM experienced in management and technical and scientific activities - The social medicine group linked to UAM-X maintains influence on reforms - Partial adhesion of the states, with resistance related to the recentralization of functions and the party political profile	Extinction of INSABI and search for expansion of coverage via IMSS-Bienestar, with recentralization of functions 2023: Implementation of IMSS-Bienestar and recentralization of the national health system 2023: Recentralization of funding via the Health Services Contribution Fund (FASSA, acronym in Spanish) and the Health Welfare Fund (Fonsabi, acronym in Spanish). 2023: End of the COVID-19 pandemic in Mexico.

GDP: gross domestic products; INSABI: Institute of Health for Welfare; PEMEX: Petróleos Mexicanos; UAM-X: Metropolitan Autonomous University - Xochimilco; UNAM: National Autonomous University of Mexico.

Source: prepared by the authors based on various sources. Health financing data obtained from the World Health Organization (https://apps.who.int/nha/database/, accessed on Apr/2026).

Although out-of-pocket spending as a share of total health expenditures declined from 52.2% in 2000 to 40.6% in 2012, high levels of such spending persisted throughout the entire period. The proportion increased to 42.3% in 2018 and fell to 39.1% in 2022 (Box 1), a still elevated level that suggests the reforms had only limited effects in reducing household health expenditures.

### Coverage and service utilization: changes within a segmented system

To better understand the effects of health reforms in Mexico, indicators of coverage and service utilization were analyzed, as well as possible implications of these changes for health system segmentation and regional inequalities.

**Figure 1:**
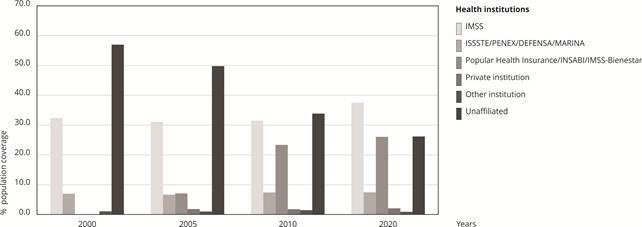
Evolution of percentage population coverage according to health institutions. Mexico, 2000, 2005, 2010 and 2020.

Figure 1 presents the proportion of the population covered by health institutions from 2000 to 2020. The indicators show a reduction in the proportion of people without institutional affiliation, from 57% in 2000 to 26.2% in 2020. IMSS remained the main institution within the system, covering more than 30% of the population throughout all years analyzed. The expansion of formal coverage for the population without social security is particularly noteworthy. The combined proportion of the population covered by Popular Health Insurance, INSABI, or IMSS-Bienestar increased from 7.1% in 2005 to 26.1% in 2020, representing a 268% increase. No significant changes were observed in the specific schemes for public servants or in the share of the population linked to private institutions. The data suggest that, despite the weaknesses identified in the implemented reforms, coverage within the SNS expanded.

IMSS: Mexican Social Security Institute; ISSSTE: Institute of Social Security and Services for Goverment Workers; MARINA: Secretariat of the Navy; PEMEX: Petróleos Mexicanos. Source: Instituto Nacional de Estadística y Geografía ^34^. Note: the sum of the percentages may be greater than one hundred percent due to the population that declared affiliation with more than one institution. The figures do not include populations classified as “unspecified” and those affiliated with the IMSS-Bienestar Program, aimed at the rural population, as data were only available for the last year of the series.

Coverage by federative entity is presented in Table 1. Even with the expansion of Popular Health Insurance and later INSABI, states such as Hidalgo, Chiapas, and Michoacán still showed proportions of uninsured individuals above 30%. Other states, such as Nuevo León, Coahuila, and Sonora, recorded higher levels of coverage via social security institutions.

**Table 1:** Percentage population coverage by health institutions, by state. Mexico, 2000, 2010, and 2020.

Federative Entity	IMSS (%)			ISSSTE, PEMEX, DEFENSA, MARINA (%)			Popular Health Insurance and IMSS-Bienestar (%)			Not covered (%)		
	2000	2010	2020	2000	2010	2020	2000	2010	2020	2000	2010	2020
Aguascalientes	48.0	44.8	54.8	7.8	7.7	7.0	-	25.6	19.1	43.4	21.1	18.4
Baja California	44.7	43.7	52.9	5.2	7.2	6.7	-	15.0	13.7	38.1	28.8	22.2
Baja California Sur	42.4	41.9	51.2	16.9	14.8	14.4	-	18.3	17.5	39.2	22.8	16.2
Campeche	27.4	29.5	31.0	11.6	10.9	10.4	-	36.7	35.3	60.5	22.2	21.9
Coahuila de Zaragoza	61.8	56.4	66.3	7.1	7.0	6.6	-	10.8	5.8	28.4	21.9	19.0
Colima	37.7	40.1	49.5	8.3	8.3	8.4	-	32.8	24.6	48.2	17.3	16.8
Chiapas	11.7	13.0	12.1	5.1	5.3	5.4	-	37.3	45.8	77.8	41.7	32.7
Chihuahua	49.2	45.2	54.6	4.5	4.7	5.7	-	17.8	18.4	39.3	23.5	15.3
Mexico City	36.6	34.3	42.1	14.4	13.6	13.5	-	10.6	13.1	45.9	33.6	27.2
Durango	38.3	35.0	40.6	10.8	10.5	11.0	-	22.0	22.1	49.2	29.8	25.2
Guanajuato	28.7	28.7	36.6	5.3	5.6	5.5	-	34.5	35.4	64.5	29.6	20.7
Guerrero	12.2	12.2	14.3	8.3	8.7	9.7	-	31.4	49.3	78.1	45.7	25.2
Hidalgo	21.7	19.7	24.1	7.6	7.8	8.6	-	37.1	35.6	69.6	33.8	30.1
Jalisco	41.2	41.4	49.7	3.1	3.1	3.1	-	17.0	14.5	53.8	34.5	29.4
State of Mexico	31.2	29.5	35.2	5.7	7.7	7.8	-	17.5	20.3	54.8	40.4	33.4
Michoacán de Ocampo	20.6	21.7	25.4	5.7	6.1	6.5	-	25.0	28.9	72.0	44.4	37.6
Morelos	26.9	26.3	31.3	7.6	7.5	8.3	-	28.1	31.4	61.7	35.3	28.0
Nayarit	31.0	30.7	37.6	10.0	10.7	11.0	-	35.3	28.0	58.5	22.8	22.1
Nuevo León	59.6	56.6	62.2	4.1	4.1	4.3	-	10.7	9.7	31.2	20.3	18.5
Oaxaca	15.8	15.1	14.1	7.0	7.4	8.1	-	32.6	46.0	76.0	43.1	29.4
Puebla	20.1	19.8	22.0	4.0	5.2	5.3	-	22.8	41.3	71.4	49.3	29.2
Querétaro	40.8	39.7	47.3	4.8	4.5	4.4	-	27.7	24.8	52.6	25.2	20.5
Quintana Roo	38.0	39.1	44.5	8.3	7.5	6.6	-	19.2	20.7	51.5	29.8	25.2
San Luis Potosí	31.1	32.7	39.6	6.1	6.1	6.3	-	33.2	34.1	61.2	25.9	17.4
Sinaloa	45.3	41.5	50.1	8.2	8.6	9.3	-	24.4	20.8	45.4	24.5	19.0
Sonora	45.8	44.4	53.6	7.0	10.8	10.1	-	17.3	15.8	41.7	25.0	18.6
Tabasco	14.5	14.1	20.8	9.9	11.6	11.2	-	44.7	33.1	69.5	25.2	31.4
Tamaulipas	40.9	38.3	47.3	10.4	9.0	9.0	-	23.8	20.9	46.5	22.5	20.2
Tlaxcala	23.2	18.1	24.1	6.6	6.5	6.8	-	35.1	39.6	68.8	37.8	26.6
Veracruz de Ignacio de la Llave	23.7	24.8	28.1	7.2	7.3	7.4	-	25.3	35.1	68.1	39.9	27.6
Yucatán	39.3	38.9	42.2	5.9	5.6	6.1	-	28.8	27.1	53.9	24.1	21.8
Zacatecas	26.0	26.6	32.5	6.6	7.1	7.3	-	34.6	39.5	66.3	30.5	20.1
National total	32.3	31.5	37.5	7.0	7.4	7.5	-	23.3	26.1	57.0	33.8	26.2

DEFENSA: Department of National Defense; IMSS: Mexican Social Security Institute; MARINA: Secretariat of the Navy; ISSSTE: Institute of Social Security and Services for Goverment Workers; PEMEX: Petróleos Mexicanos.

Source: Instituto Nacional de Estadística y Geografía ^34^.

**Figure 2:**
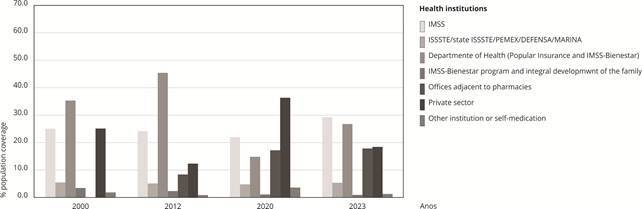
Evolution of health service utilization by type of establishment, according to health institutions. Mexico, 2000, 2012, 2020, and 2023.

Regarding service utilization, ENSANUT data suggest both continuities and changes over time. Figure 2 shows that, in both 2000 and 2012, only about 30% of the population reported seeking care from services linked to corporate social security institutions when healthcare was needed. The proportion of individuals seeking public services provided by the health secretariats increased between these two years, during the expansion of Popular Health Insurance, from 35.3% in 2000 to 45.4% in 2012. During the same period, the proportion of respondents reporting use of the private sector decreased from 25.1% to 12.4%. However, it is important to note that, in 2012, the survey began to include visits to private offices adjacent to pharmacies (CAF, acronym in Spanish), which accounted for 8.4% of respondents.

IMSS: Mexican Social Security Institute; ISSSTE: Institute of Social Security and Services for Goverment Workers; MARINA: Secretariat of the Navy; PEMEX: Petróleos Mexicanos. Source: National Institute of Public Health ^35^. The following question was asked: “What is the health service/institution that you usually look for when you have/had a health need?” Note: populations classified as “unspecified” and “does not know” were not included in the figure; the IMSS-Bienstar Program refers to the program aimed at the rural population, and DIF refers to the National System for Integral Family Development (federal, state, and municipal), which provides basic services also linked to IMSS; the Ministry of Health corresponds to the infrastructure made available for Popular Insurance and later IMSS-Bienestar as a health institution; information on “offices adjacent to pharmacies” was not included in the 2000 survey; information on “did not receive care” was not included in the 2020 and 2023 surveys.

In the 2020 survey, changes can be observed in these patterns of healthcare-seeking behavior, alongside the persistence of high demand for private services (including CAFs and private medical offices). The proportion of the population seeking services from social security institutions showed a slight decline (from 30.6% to 26.8%), while the proportion reporting use of public services experienced a sharper reduction (from 35.3% to 14.8%). In contrast, the use of CAFs increased (from 8.4% to 17.2%), as did the use of the private sector overall (from 25.1% to 36.3%); together, these categories accounted for more than 50% of responses. This was the first year of the COVID-19 pandemic, during which the population may have avoided public healthcare services and sought individual private care alternatives. At that time, the Mexican health system was undergoing the implementation of INSABI, which may also have influenced respondents’ answers.

In 2023, following the most critical phase of the public health emergency, new changes were observed: the use of public services linked to the corporate social security institutions increased (reaching a combined 34.6%), as did the use of public services provided by health secretariats/IMSS-Bienestar, while reliance on the private sector declined. However, the use of CAFs continued to rise (to 17.8%). These clinics provide outpatient care, particularly medical consultations, paid for out of pocket by users (averaging 20-30 pesos), and are attached to and affiliated with private pharmacies, which are often responsible for their management ^28^.

Service utilization by federative entity, presented in Table 2, reveals substantial territorial variation in the use of public and private services, with different combinations across states. States in which IMSS recorded the greatest expansion in coverage (Table 1) were also those showing the largest increases in the use of IMSS services: Aguascalientes, from 38.8% to 70.8%; Colima, from 33.0% to 78.7%; and San Luis Potosí, from 22.1% to 67.4%. High utilization of Popular Health Insurance/IMSS-Bienestar services was observed in states such as Guerrero, Hidalgo, and Chiapas, which are characterized by lower economic dynamism and fewer resources. In some states, such as Coahuila, high use of IMSS services coexisted with low utilization of other public services and high use of CAF (22.5%). Other states showed low IMSS utilization and high reliance on CAFs, such as Campeche (20.2%) and Michoacán (30.6%).

**Table 2:** Percentage of healthcare service utilization by type of facility, according to health institutions and by state. Mexico, 2000 and 2023.

Federative Entity	IMSS (%)		ISSSTE, PEMEX, DEFENSA, MARINA (%)		Department of Health (Popular Insurance and IMSS-Bienestar) (%)		IMSS-Bienestar program and integral development of the family (%)		Private sector (%)		Offices adjacent to pharmacies (%)		Other institution (%)	
	2000	2023	2000	2023	2000	2023	2000	2023	2000	2023	2000	2023	2000	2023
Aguascalientes	38.8	70.8	5.1	0.0	27.0	9.2	0.2	1.5	24.4	3.1	-	15.4	1.6	0.0
Baja California	37.5	45.6	5.9	3.5	14.8	0.0	0.4	0.0	35.2	33.3	-	14.0	2.4	3.5
Baja California Sur	34.5	46.2	16.0	25.0	28.3	1.9	0.0	0.0	17.6	9.6	-	17.3	0.5	0.0
Campeche	17.7	39.3	7.3	7.9	48.6	25.8	5.8	0.0	15.7	5.6	-	20.2	2.1	0.0
Coahuila de Zaragoza	54.8	51.9	5.0	6.1	13.1	9.6	0.7	0.0	21.6	9.2	-	22.5	2.2	0.7
Colima	33.0	78.7	7.8	9.8	33.9	6.6	0.1	0.0	20.2	3.3	-	1.6	1.8	0.0
Chiapas	10.4	41.2	4.6	0.0	47.3	29.8	13.1	0.0	16.5	18.4	-	10.5	2.4	0.0
Chihuahua	39.8	47.7	4.2	7.0	19.0	23.7	0.4	2.0	30.7	9.3	-	8.2	3.5	2.2
Mexico City	25.6	32.8	10.0	10.2	26.5	17.0	0.1	0.5	32.1	18.7	-	18.6	2.6	1.7
Durango	29.3	50.0	6.9	9.0	33.6	16.7	7.0	0.0	19.9	7.7	-	16.7	0.8	0.0
Guanajuato	22.2	25.7	3.6	4.0	25.7	30.5	0.0	0.0	42.7	16.7	-	22.0	1.4	0.9
Guerrero	9.8	3.0	4.4	8.0	59.8	65.0	0.1	0.0	22.2	19.0	-	5.0	0.4	0.0
Hidalgo	14.5	18.0	3.2	5.1	46.0	38.5	11.7	1.3	21.8	22.0	-	14.4	0.7	0.5
Jalisco	30.8	41.5	1.8	2.4	30.6	22.9	0.0	0.2	28.5	17.7	-	13.2	3.1	1.9
State of Mexico	26.6	21.6	3.5	5.0	32.3	24.3	0.1	0.4	29.6	23.2	-	23.2	3.6	2.1
Michoacán de Ocampo	11.1	15.3	3.9	7.7	35.3	26.0	2.7	1.7	40.3	17.9	-	30.6	1.3	0.4
Morelos	21.1	21.7	7.2	4.9	38.5	27.5	0.1	0.1	29.2	22.1	-	21.2	0.8	1.8
Nayarit	23.4	35.7	6.6	4.8	44.6	25.6	2.6	5.3	18.3	16.0	-	11.2	1.4	1.3
Nuevo León	46.1	37.6	2.8	4.7	19.1	25.9	0.0	0.0	24.1	15.3	-	14.1	3.2	2.4
Oaxaca	6.4	7.6	6.0	12.4	57.2	52.4	15.6	0.0	10.6	21.9	-	2.9	0.9	2.9
Puebla	12.9	14.2	3.9	2.3	36.1	32.8	11.3	1.9	30.1	38.1	-	9.7	1.6	1.0
Querétaro	32.8	21.6	2.6	1.8	33.9	32.0	0.1	0.0	25.0	24.6	-	18.0	1.6	1.8
Quintana Roo	22.9	32.1	5.4	3.9	40.6	20.1	0.2	0.1	27.1	15.8	-	27.1	0.4	0.7
San Luis Potosí	22.1	67.4	3.6	2.2	35.5	10.1	6.4	1.1	26.7	6.7	-	12.4	1.4	0.0
Sinaloa	36.2	39.7	6.2	6.0	24.2	19.1	3.2	1.1	24.1	10.5	-	22.3	3.2	1.3
Sonora	31.7	42.9	8.6	6.5	29.2	20.1	0.1	1.3	23.8	13.4	-	13.7	2.8	1.7
Tabasco	9.2	11.7	6.5	17.2	58.1	37.4	0.1	0.0	17.5	12.6	-	18.0	3.3	3.0
Tamaulipas	30.6	34.2	8.1	0.0	24.1	13.7	1.1	19.2	29.2	5.5	-	27.4	3.2	0.0
Tlaxcala	19.3	20.2	4.4	5.3	42.4	37.8	0.2	1.2	29.5	23.4	-	10.5	1.6	1.6
Veracruz de Ignacio de la Llave	15.5	3.3	5.0	11.5	43.0	41.0	5.3	0.0	22.6	34.4	-	9.8	2.7	0.0
Yucatán	26.8	21.1	4.6	4.2	31.4	38.6	13.5	0.0	20.0	25.9	-	8.4	1.1	1.8
Zacatecas	19.0	3.9	4.4	13.2	40,7	34.2	4.6	0.0	27.2	40.8	-	7.9	0.6	0.0
National total	26.25	29.29	5.48	5.35	35.32	27.23	3.44	0.71	23.96	18.43	-	17.84	1.85	0.88

DEFENSA: Department of National Defense; IMSS: Mexican Social Security Institute; MARINA: Secretariat of the Navy; ISSSTE: Institute of Social Security and Services for Goverment Workers; PEMEX: Petróleos Mexicanos.

Source: National Institute of Public Health ^35^.

Note: populations classified as “unspecified” and “does not know” were not included in the table; the IMSS-Bienestar Program refers to the program aimed at the rural population, and integral development of the family refers to the National System for Integral Family Development (federal, state, and municipal), which provides basic services also linked to IMSS; the Ministry of Health corresponds to the infrastructure made available for Popular Insurance and later IMSS-Bienestar as a health institution; information on “private offices adjacent to pharmacies” was not included in the 2000 survey; information on “did not receive care” was not included in the 2020 and 2023 surveys.

## Discussion

The Mexican health system, which is corporative in nature, has undergone reforms in recent decades that have not overcome its institutional segmentation and fragmentation, historically associated with labor-market attachment and territorial heterogeneity. This is due to asymmetries in the coverage provided by social security institutions − which, despite the use of the term “social security”, continue to operate according to a social insurance logic tied to employment status − as well as disparities in states’ capacity to provide services. In a structurally unequal region such as Latin America, these characteristics hinder the reduction of health inequities, which tend to worsen during periods of economic crisis marked by high unemployment and labor informality ^3^ ^,^ ^10^.

The health reforms implemented during the study period, under different administrations, declared as their objectives the expansion of coverage and the reduction of private health expenditures, but differed in terms of principles and strategies adopted. Reform processes were shaped by different institutions and groups of actors, as well as by the political orientation of governments in power.

Some public and private institutions served as spaces for articulating ideas and proposals, including differing views regarding the role of the state and the private sector. The National Institute of Public Health, linked to the Ministry of Health, played a prominent role in professional training and in the development of research and surveys such as ENSANUT. Funsalud, a private organization, formulated proposals for health system reform and succeeded in placing its leaders in positions within the Ministry of Health ^4^ ^,^ ^10^. Also noteworthy was the role of National Autonomous University of Mexico (UNAM) and the Metropolitan Autonomous University - Xochimilco (UAM-X) in critical thinking, professional training, and the education of managers working within the health system ^3^ ^,^ ^10^.

Since the 1980s, two groups have stood out in shaping health policy agendas. One is the “new public health” group, led by Julio Frenk, initially linked to the National Institute of Public Health, Funsalud, and UNAM. The other group, identified with the Latin American Social Medicine movement, was led primarily by Asa Cristina Laurell, professor at UAM-X, who served as Secretary of Health of the Federal District and held a leadership position at IMSS-Bienestar ^11^.

The first group influenced the reforms implemented between 2000 and 2018, during the administrations of Vicente Fox, Felipe Calderón (both from PAN), and Enrique Peña Nieto (PRI). The implementation of Popular Health Insurance led to an increase in enrollment and coverage of a limited package of interventions, which gradually expanded over time. However, inequalities across states became evident in terms of financing, management capacity, enrollment, quality, and access to services ^2^, alongside growing contracting of private services using public resources ^3^ ^,^ ^20^. In summary, this reform increased insurance coverage but reinforced system segmentation, limited the expansion of public services, and favored the private sector via incentives or weak state regulation. Out-of-pocket health expenditures remained high throughout the period, signaling limitations of one of the reform’s stated objectives: financial protection.

Although Mexico’s 2000s reform shared guidelines with Colombia’s “structured pluralism” modelsuch as emphasis on expanding coverage, separating functions, and promoting a mix of public and private providers ^5^ − important differences remained. In the Mexican case, the coverage arrangement for formal workers through the corporate institutions was preserved, while Popular Health Insurance was adopted to expand coverage for poor and informal workers by means of a nationally defined package of services.

Beginning in 2018, with the election of López Obrador, proposals associated with the Social Medicine group gained greater prominence, particularly those advocating for a stronger national state, recentralization of health functions, the dismantling of Popular Health Insurance, greater coordination among public institutions and services, and the containment of private sector expansion. The creation of INSABI as a strategy to expand coverage and access for the uninsured population represented an effort toward universalization, but it encountered structural constraints, institutional fragility, conflicts among Federative Entities and political groups (both within the government and social security institutions), as well as the challenges posed by the public health emergency.

IMSS-Bienestar represented a reorientation of the government’s strategy to expand access to healthcare, emphasizing the recentralization of management, coordination among existing institutions and services, and the adoption of a PHC-based model supported by public services provided by IMSS- Bienestar, IMSS, and ISSSTE. However, like previous strategies, its implementation depends on adherence of both the states (which has thus far been only partial) and social security institutions, given the expectation of coordination among institutions and services.

Regarding the private sector, it is important to highlight the alarming growth of physician offices adjacent to pharmacies and their use by the Mexican population ^28^ ^,^ ^29^ ^,^ ^30^. In 2021, there were approximately 18,000 such clinics in the country, providing an average of 325,000 medical consultations per day ^31^. The proportion of Mexicans who reported usually seeking this service more than doubled between 2012 and 2023, reaching 17.8% ^30^. This is a concerning finding, particularly when considered alongside the country’s persistently high level of out-of-pocket health expenditures (39.1% in 2022, Table 1) ^32^.

The use of CAFs predominates among individuals without social security coverage and those in precarious employment conditions, who seek medical care when health problems prevent them from working. Demand for these services is driven by rapid access and low cost of consultations, which allow patients to obtain a medical prescription and return to work quickly ^32^. Since these clinics operate as healthcare services without a clearly defined formal status and are attached to commercial establishments, they have expanded outside the framework of the national health system ^28^ ^,^ ^30^ and under weak regulation, lacking clear rules regarding their operation and oversight.

Demand for these services increased during the pandemic due to the saturation of healthcare services and the risk of infection in hospitals ^3^ ^,^ ^30^, and it remained high in the post-pandemic period, especially among lower-income urban families ^10^. Major concerns include precarization of medical work, uncertainty regarding the quality of services provided, lack of records on the clinics and the care delivered, and risk of excessive medication prescribing ^10^ ^,^ ^28^.

The proliferation of these clinics hinders the strengthening of an integrated public health system based on PHC, while exacerbating the commodification and precarization of care. These clinics provide fragmented, demand-driven basic healthcare services that do not replace the type of care that should be ensured via PHC according to the principles of comprehensiveness, territoriality, continuity, and service integration. The State has been either negligent or weak in regulating these clinics. There is no precise information regarding the number of consultations, healthcare professionals, prescribed medications, health complaints, or the geographic distribution of these establishments.

In this sense, elements inherited from health reforms implemented in previous decades − such as the persistent segmentation of the system, public underfunding, and the expansion of private services − are in line with the movement toward universalizing access to healthcare proclaimed by recent reforms. The persistence of high out-of-pocket expenditures, combined with the growing use of private clinics, suggests the limitations of the reforms implemented over recent decades in strengthening the public system, expanding access to public services, and ensuring financial protection in healthcare.

Regarding study limitations, the small number of interviews did not enable a qualitative analysis of responses, despite their importance as a complementary source for understanding the context of the reforms, given the expertise of the key informants interviewed. The decision to use data from two reliable national sources (the census and the ENSANUT survey) restricted the analysis to the years and indicators available in those databases, which were used as proxies for institutional coverage and service utilization, resulting in a limited analysis of inequalities within the country. There are also limitations in analyzing coverage and access data over time, for example due to the absence of mandatory enrollment under INSABI or IMSS-Bienestar. The expansion of physician offices adjacent to pharmacies requires specific studies exploring changes in the public-private configuration of healthcare in the country. Finally, the recent and only partially implemented nature of IMSS-Bienestar makes it difficult to assess its outcomes.

On April 7, 2026, the President of Mexico announced the creation of a Universal Health Service, with the gradual enrollment of the entire population, who will be able to access all healthcare services linked to the country’s different institutions, regardless of employment status ^33^. The implementation of this reform, which will require further study in the coming years, may represent an important step toward universalizing access and overcoming the corporate and segmented logic that has historically characterized the Mexican health system.

## Conclusion

The reforms implemented in the Mexican health system over recent decades, under governments with different political orientations, have not overcome the historical legacy of segmentation by population groups, organizational fragmentation, and health inequalities. To date, access to healthcare in Mexico remains stratified according to employment status and ability to pay, resulting in different forms of citizenship within the same society. A universal health system accessible to the entire population has not been established, nor have structural changes been implemented to universalize access to services linked to the social security institutions.

Health reforms in the country were shaped by the political orientation of successive governments and by disputes among projects advanced by different groups. Despite expanding formal coverage of a restricted package of services, Popular Health Insurance reinforced segmentation, deepened inequalities, promoted the expansion of private providers financed with public resources, and failed to achieve its stated goals of universal coverage and financial protection.

Reforms implemented from 2018 onward, under center-left governments, sought to universalize access with greater equity, but encountered structural, institutional, and political obstacles that led to a reorientation of strategies. These reforms have been characterized by movements toward the recentralization of responsibilities and resources within the Federal Government, as well as efforts to promote greater coordination with corporate institutions and among healthcare services.

Throughout successive reforms, significant challenges to the universalization of healthcare access in Mexico have persisted, including insufficient public funding; limitations in infrastructure and healthcare workforce in many states; resistance and risk of non-adherence by states and social security institutions to reform proposals; federal and partisan political conflicts; expansion of private services under weak regulation; and marked territorial and social inequalities.

## Data Availability

The sources of information used in the study are indicated in the body of the article.
