# Supplementary material for: Health policies and reforms in Mexico (2000-2024): segmentation and challenges for universal rights
Source: Cad Saude Publica. 2026 Aug 10;42(Suppl 1):e00278225. doi: 10.1590/0102-311XEN278225 (PMC13460892; doi:10.1590/0102-311XEN278225)
Supplement: Translation

# Translation: Políticas e reformas de saúde no México (2000-2024): segmentação e desafios para o direito universal

**DOI:** 10.1590/0102-311XPT278225

**Authors:** Eduarda Ferreira dos Anjos, Oliva López-Arellano, Elzo Pereira Pinto, Cristiani Vieira Machado

**Affiliations:** 1 Escola Nacional de Saúde Pública Sergio Arouca, Fundação Oswaldo Cruz, Fiocruz, Rio de Janeiro, Brasil.; 2 Instituto Gonçalo Moniz, Fundação Oswaldo Cruz, Fiocruz, Salvador, Brasil.; 3 Universidad Autónoma Metropolitana, Ciudad de México, México.

**Keywords:** Cobertura de Serviços de Saúde, Acesso Universal aos Serviços de Saúde, Utilização de Serviços, Parcerias Público-Privadas, América Latina

## Abstract

O sistema de saúde mexicano é historicamente segmentado, baseado em um seguro social para trabalhadores formais e em ações de saúde pública para a população em geral. O artigo caracteriza as reformas de saúde no México entre 2000 e 2024, considerando-se a trajetória das políticas de saúde, a cobertura e a utilização do sistema de saúde. A pesquisa envolveu revisão bibliográfica, análise documental e de dados secundários, complementadas por entrevistas com informantes-chave. Observaram-se diferentes momentos de reforma, influenciados por distintos grupos e orientações políticas dos governos. Entre 2003 e 2018, a implantação de um Seguro Popular de Saúde voltado a trabalhadores informais e pobres, com oferta limitada de serviços, levou ao aumento da cobertura, mas reiterou segmentação e desigualdades. Entre 2018 e 2024, sob governos de centro-esquerda, extinguiu-se o seguro popular e foram adotadas estratégias de reforma que envolvem a centralização de funções no Governo Federal e esforços de integração entre instituições e serviços públicos. Os resultados mostraram, ainda, a expansão da utilização de consultórios privados adjacentes a farmácias. Conclui-se que, no período, as reformas adotadas não superaram o legado de segmentação por clientelas, fragmentação organizacional e desigualdades em saúde. Apesar do anúncio em abril de 2026 de reformas no caminho da universalização da saúde, persistem como desafios o insuficiente financiamento público, limitações de infraestrutura e de força de trabalho de saúde nos estados, resistências dos estados e das instituições previdenciárias, conflitos federativos e político-partidários, expansão de serviços privados sob escassa regulação e desigualdades territoriais e entre grupos sociais.

## Introdução

O México é o segundo país mais populoso da América Latina - com mais de 126 milhões de habitantes - e a segunda maior economia da região. Apesar disso, em 2020, cerca de 43,9% da população viviam em situação de pobreza, 22,5% sem acesso à alimentação adequada e 28,2% sem acesso assegurado a serviços de saúde. O país é extenso, marcado por diversidade e desigualdades em diferentes dimensões: territoriais, socioeconômicas, étnico-raciais. É uma federação composta por 32 estados e 2.475 municípios, o que impõe desafios à coordenação das políticas públicas. É uma república presidencialista, com uma história política marcada por um longo período de um único partido no poder e alternância de governos nas últimas duas décadas, que se expressou na orientação das políticas de saúde ^1^.

O sistema de saúde mexicano é historicamente caracterizado por segmentação de clientelas, fragmentação organizacional e desigualdades na oferta, cobertura e resultados sanitários ^2^. Sua organização tem como marcos a criação da Secretaria de Saúde e Assistência (SSA) − posteriormente denominada Secretaria de Saúde, equivalente ao Ministério da Saúde no Brasil − e do Instituto Mexicano de Seguridade Social (IMSS) em 1943. Ao longo dos anos foram criadas outras instituições de seguridade social, responsáveis pela gestão de aposentadorias e pensões e oferta de serviços de saúde aos trabalhadores formais: o Instituto de Segurança Social e Assistência Social dos Funcionários Públicos (ISSSTE, acrônimo em espanhol, 1960), do Petróleos Mexicanos (PEMEX, acrônimo em espanhol, 1950), da Secretaria de Marina (MARINA, 1965) e da Secretaria de Defesa Nacional (SEDENA, 1976) ^3^.

Em que pese a sua importância, até os anos 1980 as instituições previdenciárias cobriam cerca de 50% da população, ao passo que milhões de mexicanos sem seguridade social dependiam de serviços públicos de cobertura restrita, desigualmente distribuídos entre os estados. Ao longo das décadas de 1980 e 1990, sob governos do Partido Revolucionário Institucional (PRI), reformas neoliberais afetaram a economia, as políticas sociais e de saúde, com descentralização de responsabilidades e serviços para os estados, sob condições institucionais e de financiamento inadequadas ^4^.

A partir dos anos 2000, com a eleição de governos de distintas orientações políticas, o sistema de saúde mexicano passou por reformas que buscaram expandir a cobertura da população sem seguridade social, sem romper com o arranjo corporativo dos institutos responsáveis pela previdência e saúde. Em um contexto de reformas neoliberais, foi implantado um Seguro Popular de Saúde sob o argumento de alcançar a cobertura universal em saúde, em sintonia com recomendações de agências internacionais para reformas na América Latina ^5^. A partir de 2018, uma mudança de governo levou à extinção desse seguro e a reformas institucionais com orientação distinta ^6^ ^,^ ^7^.

Este artigo caracteriza as políticas de saúde no México entre 2000 e 2024, período marcado por disputas de projetos e reformas do sistema de saúde de diferentes sentidos. Busca-se analisar continuidades e mudanças no contexto e nas agendas de reformas ao longo do tempo, bem como explorar seus efeitos sobre a segmentação institucional da cobertura em saúde e a utilização de diferentes tipos de serviços de saúde.

## Metodologia

Realizou-se um estudo de caso do sistema de saúde mexicano, cuja escolha deveu-se à importância do país na América Latina, às pressões das agendas neoliberais sobre a saúde desde os anos 1980 e ao dinamismo das reformas em saúde nas últimas décadas. O período do estudo (2000-2024) compreende transições de poder entre governos de diferentes orientações políticas ^7^.

O referencial analítico do estudo apoia-se na literatura sobre análise de políticas de saúde na América Latina, considerando que os sistemas de saúde resultam de processos históricos em que as instituições influenciam as estratégias dos atores, trajetória e conteúdo das políticas, em cada contexto. Tais políticas resultam de normas institucionais, processos decisórios, ações governamentais e atuação de grupos de interesse ^8^. A análise de políticas setoriais requer a compreensão das regras, estruturas, mobilização de recursos e de agentes, considerando-se legados históricos e decisões do presente ^9^.

Os eixos analíticos do estudo foram: trajetória histórico-institucional das políticas de saúde; cobertura e utilização do sistema de saúde por segmentos da população.

As políticas de saúde no período são analisadas em três momentos: criação e vigência do Seguro Popular de Saúde (2000-2018); criação e vigência do Instituto de Saúde para o Bem-Estar (INSABI, 2019-2022); e sua substituição pelo IMSS-Bienestar (2023-atual). A caracterização da cobertura do sistema foi realizada para o conjunto do período, considerando-se os dados nacionais e por estados.

A pesquisa envolveu revisão bibliográfica de livros e periódicos nas bases Biblioteca Virtual de Saúde; Embase; PubMed; Scielo; Web of Science; e Scopus e análise de documentos oficiais de 2000 a 2024, como legislação, relatórios e resoluções.

A cobertura e a utilização do sistema de saúde foram sistematizadas com base em dados secundários oficiais de acesso público, na escala nacional e por estados. Os dados de cobertura por tipo de filiação institucional da população foram obtidos do Censo do Instituto Nacional de Estatística e Geografia (INEGI) para os anos de 2000, 2005, 2010 e 2020. Os dados de utilização foram extraídos da pergunta “serviços/instituições de saúde usualmente procurados em caso de necessidade de saúde”, da *Pesquisa Nacional de Saúde e Nutrição* (*Encuesta Nacional de Salud y Nutrición*, ENSANUT) - pesquisa por amostragem - para os anos de 2000, 2012, 2020 e 2023. Foram realizadas análises descritivas, com cálculo de percentuais e elaboração de tabelas no Microsoft Excel (https://products.office.com/).

Durante duas semanas, em abril de 2024, realizou-se trabalho de campo no México, com observação direta em serviços de saúde públicos e privados, participação em reuniões técnicas relativas à reforma em curso e interação com pesquisadores de universidades do país, profissionais de saúde e gestores municipais no Distrito Federal e no interior. De modo complementar, realizaram-se quatro entrevistas semiestruturadas com informantes-chave, sendo três presenciais durante o trabalho de campo e uma on-line, orientadas por um roteiro com questões sobre os contextos e os processos de reforma. Os entrevistados foram escolhidos por seu conhecimento e envolvimento nas reformas do sistema de saúde no período, sendo: dois pesquisadores do tema que atuaram como gestores no Distrito Federal; um pesquisador do tema, do Instituto Nacional de Saúde Pública; e um representante de organismo internacional que acompanhou a reforma recente. 

O projeto foi aprovado pelo Comitê de Ética em Pesquisa da Fundação Oswaldo Cruz (Fiocruz, CAE 18083719.9.0000.5240).

## Resultados

### O Seguro Popular de Saúde: em busca da cobertura universal (2000-2018)

O México constituiu um caso singular na América Latina de adoção de um Seguro Popular de Saúde, voltado à cobertura de pessoas pobres e trabalhadores informais, para viabilizar o acesso a um pacote restrito de serviços e medicamentos. Sem romper com o arranjo corporativo de cobertura em saúde dos trabalhadores formais por meio dos institutos previdenciários, a implantação do Seguro Popular de Saúde visaria a atingir a cobertura universal e evitar o empobrecimento das famílias em decorrência de elevados gastos privados em saúde por desembolso direto.

A experiência do Seguro Popular de Saúde situa-se no contexto da ascensão ao poder do Partido Acción Nacional (PAN), de direita. Vicente Fox foi eleito Presidente para o sexênio 2000-2006, ao derrotar o candidato do PRI, partido que havia ocupado o Governo Federal por 71 anos ^4^. Seu governo seria marcado por uma agenda econômica liberal, flexibilização trabalhista, terceirização de serviços da seguridade social e reformas na saúde ^10^.

Na saúde, foi nomeado como ministro Júlio Frenk, ex-diretor do Instituto Nacional de Saúde Pública (INSP, 1987-1992), consultor do Banco Mundial (1995-1996) e vice-presidente executivo da Fundação Mexicana para a Saúde (Funsalud, acrônimo em espanhol; 1995-1998) ^10^ ^,^ ^11^. As reformas implantadas visaram a construir um Sistema de Proteção Social em Saúde (SPSS). Destacam-se como estratégias: mudanças legais ^12^ voltadas à sustentabilidade financeira, ênfase na eficiência, a criação do Seguro Popular de Saúde e um novo movimento de descentralização administrativa, com a assunção pelos estados de responsabilidades de gestão do Seguro Popular de Saúde, incluindo recebimento de recursos federais, contrapartida financeira, cadastramento de famílias, gerência direta ou contratação dos serviços de saúde. As políticas econômicas e administrativas mantiveram a contenção salarial e a alta informalidade laboral, com prejuízos para um sistema de saúde corporativo ^10^ ^,^ ^13^.

Para regular a aplicação da Lei Geral de Saúde (LGS), criou-se a Comissão Federal para Proteção contra Riscos Sanitários (Cofepris), com autonomia técnica, operativa e administrativa ^12^. Nesse período, houve aumento de agências reguladoras no México que, contudo, não significou melhor gestão dos recursos, eficiência financeira e protagonismo do papel do Estado ^13^. A LGS de 2001 determinou a possibilidade de contratos plurianuais, promovendo parcerias público-privadas por meio de acordos legais que podem ter vigência maior que um exercício fiscal e permitem a compra ou aluguel de bens ou serviços privados por parte do Estado ^10^ ^,^ ^14^. Isso levou à contenção de investimentos em infraestrutura própria e ao aumento da participação privada via terceirização de serviços ^1^. A Funsalud, associação de empresários sem fins lucrativos, propôs a criação de um fundo privado para programas de saúde pública e influenciou a criação do Sistema de Proteção Social em Saúde, que teve como braço operacional o Seguro Popular de Saúde ^7^.

O Seguro Popular de Saúde teve três fases: lançamento (2000-2006), consolidação (2006-2012) e declínio (2012-2018) ^1^. A sua implantação, dependente de adesão voluntária dos entes federados, começou em cinco estados em 2001 e alcançou os 32 estados em 2004. A Cidade do México foi o último ente a aderir, pela resistência do chefe de governo, Andrés Manuel López Obrador, na época filiado ao Partido da Revolução Democrática (PRD) ^15^, de oposição.

A Comissão Nacional de Proteção Social em Saúde e os Regimes Estaduais de Proteção Social à Saúde, nos níveis federal e estadual, foram responsáveis pela execução do sistema, garantindo o financiamento e a prestação de serviços, empregando a estratégia dos contratos plurianuais ^4^ ^,^ ^16^.

O financiamento do Seguro Popular de Saúde era tripartite, composto por *per capita* federal, estadual e contribuições das famílias/indivíduos assegurados, proporcionais à renda, com isenção mediante comprovação de incapacidade de pagamento. O número de famílias filiadas definia o per capita dos estados ^17^. Em relação à oferta, a cesta de serviços essenciais coberta era definida pelo Catálogo de Benefícios Médicos, que consistia em 78 intervenções de saúde (2002-2003) e 191 medicamentos e vacinas. Esse rol foi ampliado e, em 2019, o Catálogo Universal de Serviços de Saúde (CAUSES) cobria 294 intervenções e 670 medicamentos, vacinas e insumos ^18^. A supervisão da prestação e da garantia da operacionalização do CAUSES ficava a cargo dos estados, que assumiam a responsabilidade de articulação e contratação de prestadores de serviços de saúde para atenção às pessoas seguradas ^19^. Mesmo com a expansão, tais serviços ainda eram inferiores aos ofertados pelo IMSS e ISSSTE, com desigualdades entre prestadores.

O período de consolidação do Seguro Popular de Saúde ^1^ (2006-2012) correspondeu ao mandato de Felipe Calderón, também do PAN, cuja eleição foi marcada por pequena diferença de votos em relação a Andrés Manuel López Obrador, o que desencadeou polarização política e contestação dos resultados ^5^. As reformas adotadas buscavam viabilidade financeira, otimização de recursos e capacidade de gestão ^10^. Assumiu-se o compromisso de redução da mortalidade materna e em menores de cinco anos, além do combate a doenças como HIV/aids, malária e tuberculose. O Ministério da Saúde definiu indicadores para monitorar o desempenho dos serviços estaduais e adotou um modelo de financiamento parcialmente baseado em avaliação de desempenho e resultados ^19^.

O Seguro Popular de Saúde expandiu afiliações, intervenções do CAUSES e orçamento até 2010, contando com serviços ambulatoriais das secretarias de saúde estaduais e privados contratados, assim como serviços especializados e hospitais majoritariamente privados ^2^. Nos primeiros anos, houve êxito na priorização das famílias de menor renda e redução do gasto por desembolso direto com medicamentos ^6^. Contudo, os investimentos no IMSS e ISSSTE ainda eram superiores, com um gasto governamental per capita da seguridade social superior ao do Seguro Popular de Saúde ^20^.

As medidas reformistas mantiveram a orientação liberal e buscaram melhorar a capacidade de gestão do sistema, viabilidade financeira e aproveitamento dos recursos. Em 2012, a Comissão de Proteção Social em Saúde anunciou que o México havia alcançado a cobertura universal em saúde, com cerca de 52 milhões de pessoas filiadas ao Seguro Popular de Saúde ^2^ ^,^ ^3^. Contudo, houve controvérsias sobre os resultados alcançados, apontando que 25% da população não possuía nenhum tipo de seguro de saúde ^2^.

A fase de declínio do Seguro Popular de Saúde ^7^ coincide com o retorno do PRI ao poder com o Presidente Enrique Peña Nieto (2012-2018). A promessa do governo era de modernização, transparência e combate à violência. Nesse contexto, reformas estruturais abriram espaço para o capital privado nos setores de energia, comunicação e educação ^4^.

Na saúde, a ministra nomeada foi a médica Mercedes López, que era presidente executiva da Funsalud e empenhou-se na construção de um Sistema Nacional de Saúde Universal (Sinasu). O Programa do Setor da Saúde (2013-2018) destacou três problemas: o modelo de atenção à saúde curativo e não preventivo, a incompletude na implementação de políticas multidisciplinares, e governança e arranjos financeiros desiguais ^10^.

Assim, desenhou-se em 2015 o Modelo de Atenção Integral à Saúde, que objetivou uniformizar serviços e práticas, com cuidados centrados na pessoa, otimização de recursos e infraestrutura, fortalecimento dos Sistemas Locais de Saúde e das Redes Integradas de Serviços de Saúde ^20^. Investiu-se no fortalecimento de cuidados primários custo-efetivos com projetos voltados à medicina familiar e à atenção primária. Esse modelo de organização buscava a implementação das redes e diretrizes para o acompanhamento de pessoas, especialmente com doenças crônicas, e a ampliação do acesso à atenção primária à saúde (APS), apesar de diversas outras barreiras não superadas ^10^.

A insuficiente infraestrutura para a oferta de serviços no Sinasu levou à aprovação da Lei da Associação Público-Privada, que possibilitou a construção de hospitais operados pelo IMSS, atribuindo vantagens ao setor privado ^21^. Adotaram-se mudanças como licitações conjuntas entre os subsistemas de saúde na compra de medicamentos, voltadas a custo-efetividade e transparência ^10^.

As medidas referentes ao IMSS buscavam sua consolidação financeira como instituição público-comercial ^10^. O número de pessoas cadastradas no Seguro Popular teria alcançado, em 2016, cerca de 52 milhões de pessoas ^6^, mas os seus problemas estruturais tornaram-se evidentes. O arranjo federativo do país produziu 32 subsistemas de saúde distintos, com disparidades entre os estados no financiamento per capita, na capacidade de gestão, nos recursos humanos, na infraestrutura médico-hospitalar e na oferta e na qualidade dos serviços ^2^. Persistiam altos gastos por desembolso direto sem o alcance efetivo da cobertura universal em saúde. Assim, ao final do governo Peña Nieto, período em que houve crescimento substancial do setor privado com financiamento público ^3^, estava nítido o fracasso das reformas na universalização do acesso aos serviços de saúde, na proteção financeira e na superação das desigualdades ^22^. As mudanças no IMSS e a condução do Seguro Popular de Saúde tornaram o Sinasu inviável, especialmente por questões de infraestrutura e financiamento.

### Do INSABI ao IMSS-Bienestar: em busca de um Sistema Público Universal (2018-2024)

O Partido Movimento de Regeneração Nacional (MORENA), fundado em 2011 por Andrés Manuel López Obrador, foi o primeiro partido de esquerda a assumir o governo federal após um longo período de poder de centro-direita e direita. Em sua campanha, Obrador propôs uma “quarta transformação”: erradicação da corrupção, fortalecimento do Estado e um sistema de saúde justo, com a extinção do Seguro Popular de Saúde, ao qual se referia como “*ni es seguro, ni es popular*” ^20^. O ministro da saúde foi o médico Jorge Alcocer Varela, escolhido para reformar o Sistema Nacional de Saúde (SNS) ^4^. O grupo identificado com a Medicina Social latino-americana e a luta pelo direito à saúde passa a ter mais influência, embora se identifiquem diferentes posições quanto às estratégias de reforma ao longo do governo.

O Seguro Popular de Saúde deixou de operar em 2019, substituído pelo INSABI, que contou com a adesão de 23 estados ^23^ e vigência (2019-2022) que se confundiu com o período da pandemia de COVID-19 ^24^. O INSABI foi um órgão público descentralizado cujo objetivo principal era garantir a prestação gratuita de serviços de saúde a pessoas sem seguridade social. Deveria ainda fornecer medicamentos e insumos e promover, em conjunto com a Secretaria de Saúde, ações de integração e articulação no SNS ^23^. Os princípios dessa reforma seriam a universalidade (oferta de serviços necessários às pessoas sem seguridade), acesso gratuito (prestação de serviços públicos sem taxas) e anticorrupção (centralização do controle de compras e prestação de serviços, redução da participação privada) ^20^.

Quanto ao financiamento, diferente do custeio do Seguro Popular de Saúde − em tese dividido entre governos federal, estadual e filiados, mas na prática sustentado por 80% de recursos federais e 20% estaduais − o INSABI não previa a separação de funções e tinha relação com as secretarias de saúde dos estados. O recurso do Fundo de Saúde para o Bem-estar (Fonsabi), proveniente de impostos, previa transferências diretas para os prestadores (hospitais e unidades médicas), visando à reorientação do financiamento da demanda para a oferta de serviços públicos ^20^.

A reestruturação do sistema extinguiu estruturas autônomas anteriores e incorporou responsabilidades à Secretaria de Saúde ^21^, o que gerou tensões acerca do papel das agências reguladoras. Havia também diferentes posições entre os apoiadores do governo quanto à criação de um novo instituto, em relação à possibilidade de ampliar a cobertura por meio dos institutos da Seguridade Social já existentes. Observaram-se divergências nos dados sobre número de afiliados e cobertura de serviços. O Conselho Nacional de Avaliação da Política de Desenvolvimento Social (Coneval) identificou aumento de pessoas que relataram falta de acesso a serviços de saúde, o que pode estar relacionado à extinção do Seguro Popular de Saúde e a não exigência de cadastramento no INSABI. A insuficiência de diretrizes de operação, a inexistência de serviços próprios e incertezas diante das mudanças institucionais e da emergência sanitária foram decisivas para o encerramento do INSABI ^15^ ^,^ ^20^.

O enfrentamento da pandemia de COVID-19 foi prejudicado pela fragmentação institucional e pelas dificuldades de coordenação federativa. As estratégias centraram-se na conversão de leitos hospitalares, na contratação direta de pessoal e em parcerias público-privadas ^24^. A experiência anterior com a emergência da H1N1 em 2009 foi relevante para o planejamento e a resposta a essa crise sanitária ^23^ ^,^ ^24^.

O governo contratou 50 mil médicos e enfermeiros, realizou treinamentos, adquiriu *tablets* e celulares para os profissionais. Os setores público e privado produziram insumos para a proteção individual, equipamentos e máscaras, entre outros. Mesmo com o reconhecimento tardio do governo federal da gravidade da pandemia, foram adotadas medidas de mitigação do contágio como quarentenas estratificadas conforme setores produtivos e o semáforo epidemiológico ^23^ ^,^ ^24^.

Alguns problemas no enfrentamento da pandemia foram a insuficiente realização de testes diagnósticos, escasso uso de máscaras, dificuldades de coordenação de intervenções, além das elevadas prevalências de obesidade, diabetes e hipertensão, resultando em altas taxas de letalidade por COVID-19. Ademais, gripe e pneumonia ocuparam posições principais dentre as causas de morte no período e o excesso de mortalidade geral foi elevado, sugerindo subnotificação de casos ^24^.

As medidas de austeridade do governo anterior, bem como dificuldades pregressas no sistema de compra de medicamentos, geraram interrupções na cadeia de suprimentos, insatisfação pública e protestos ^7^ ^,^ ^20^. A baixa compreensão da população quanto à reforma do INSABI e os limites de sua implementação, somados a problemas anteriores, como o subfinanciamento, levaram a uma nova reforma. Extinguiu-se o INSABI, com transferência dos seus recursos, direitos e obrigações para um novo órgão público descentralizado, o IMSS-Bienestar ^23^.

O IMSS-Bienestar iniciou um projeto-piloto em quatro estados nos quais não haveria troca de governo em 2024, visando a assegurar sua estabilidade e consolidação. O subsistema busca ofertar serviços para cerca de 53,2 milhões de mexicanos que não possuem seguridade social, nas 23 Entidades Federativas que aderiram ^4^. A estratégia envolve certa recentralização da gestão e o credenciamento dos cidadãos, para informá-los sobre seu direito à saúde. Nove estados governados pela oposição ao Governo Federal optaram por manter a gestão e a oferta de serviços por meio de suas secretarias estaduais de saúde, com capacidade variável ^1^. A adesão parcial dos estados ameaça a solidez do IMSS-Bienestar, reitera a estratificação da cobertura e do acesso e impacta o direito à saúde.

A reforma objetiva o acesso eficaz, oportuno e de qualidade aos serviços de saúde, direito ao atendimento médico-hospitalar integral, a medicamentos e demais insumos de maneira gratuita. Operacionaliza-se pelo Modelo de Atenção à Saúde para o Bem-Estar, que se aplica a todas as instituições do sistema, com foco na APS. Conta com um componente de vigilância e pode envolver convênios de colaboração com o IMSS para a utilização de recursos e equipamentos de saúde, promovendo o intercâmbio de infraestrutura e maior integração entre os serviços públicos estaduais e os do IMSS ^25^. Existe ainda, no sistema de saúde mexicano, a contratação de serviços privados, especialmente para procedimentos especializados e diagnósticos, desde as reformas dos anos 1990 ^10^.

A Junta de Governo e Administração Geral é responsável por administrar o IMSS-Bienestar e estabelecer prioridades, políticas, bases de organização e áreas que o compõem. O financiamento baseia-se em impostos, sem cota de afiliação, a fim de reduzir as desigualdades de acesso geradas no Seguro Popular de Saúde. Além das fontes financeiras federais, os governos estaduais transferem fundos diretamente para o IMSS-Bienestar, recentralizando recursos, com o intuito de evitar desvios e corrupção. Quanto à estrutura, firmou-se o Acordo Nacional para a Federalização do Sistema de Saúde para o Bem-Estar ^26^, no qual a Secretaria de Saúde nacional e as estaduais transferiram suas unidades de saúde, orçamento e pessoal para o IMSS-Bienestar, além da anexação de cinco hospitais regionais e um centro especializado.

O subsistema é organizado em três níveis de atenção, articulados em redes integradas de serviços de saúde. A APS compõe o primeiro nível, compreendendo um médico para cada 3 mil pessoas, além de cuidados de enfermagem, emergência, planejamento familiar, odontologia, nutrição, serviço social, psicologia, reabilitação e apoio diagnóstico. Os hospitais gerais do segundo nível ofertam cuidados de cirurgia geral, clínica médica, anestesiologia, pediatria, ginecologia e obstetrícia. O terceiro nível de atenção é de hospitais especializados, com mecanismos de referência e contrarreferência ^27^.

As mudanças em curso se mantêm no atual governo de Claudia Sheinbaum, primeira mulher presidente do México (2024-2030), eleita pelo MORENA com ampla diferença de votos. Alinham-se, nesse sentido, aos planos de desenvolvimento e de saúde, cujos objetivos são a consolidação de um modelo de desenvolvimento com bem-estar, justiça social e sustentabilidade. O ministro da saúde escolhido foi David Kershenobich, médico pela Universidade Nacional Autônoma do México (UNAM), que anunciou como objetivo da gestão a garantia do direito à saúde e bem-estar, articulando compromissos com a Agenda 2030 e metas mensuráveis ^27^. As características das reformas da saúde no México no período, destacando elementos do contexto, do processo político e estratégias adotadas, estão resumidas no Quadro 1.

**Quadro 1:** Contexto nacional e políticas de saúde no México, de 2000 a 2024.

MOMENTOS	CONTEXTO	PROCESSO POLÍTICO SAÚDE	AGENDA - ESTRATÉGIAS
Seguro Popular de Saúde 2000-2006 Lançamento	Governo Vicente Fox Quesada - Partido Ação Nacional (PAN, 01/Dez/2000 - 30/Nov/2006) Cenário Político-Institucional: - Transição democrática - Estabilidade macroeconômica - Forte influência do Banco Mundial na agenda de reformas - Alta popularidade Agenda econômica: - Reforma tributária econômica parcial - Privatização do setor energético e abertura de contratos privados no PEMEX - Investimento em programas de assistência à população de menor renda Financiamento da saúde: Em 2000: Gasto total saúde % do PIB: 4,2% Gasto público saúde % do PIB: 1,9% Gasto privado % do gasto total saúde: 54,8% Gasto desembolso direto % gasto total saúde: 52,2%	Júlio Frenk − Ministro da Saúde (01/Dez/2000 - 30/Nov/2006) - Liderança da “Nova Saúde Pública”, com trajetória na Funsalud, Instituto Nacional de Saúde Pública e atuação na reforma da Colômbia - Amplo poder de influência nas reformas da saúde - Influência de agendas internacionais nas reformas, com ênfase na lógica de alcance da “cobertura universal em saúde”. - Adesão gradual dos estados, influenciada por conflitos político-partidários.	Reformas gerenciais, foco na expansão da cobertura para pobres e descentralização 2001: Publicação do primeiro Programa Institucional del Instituto Mexicano del Seguro Social 2001-2006 (PIIMSS) 2001: Criação da Comissão Federal para a Proteção contra Riscos Sanitários (Cofepris) - importante instituição na descentralização do sistema de saúde 2003: Implementação do Seguro Popular de Saúde em prol do alcance da Cobertura Universal de Saúde no país 2004: Promulgação da Lei Geral de Desenvolvimento Social 2005: Criação do Conselho Nacional de Avaliação da Política Social (Coneval) - desempenhou importante papel na aceitação e consolidação do Seguro Popular 2006: Modelo Integrativo de Atenção Primária à Saúde (MIDAS) 2006: Lei Federal de Orçamento e Responsabilidade Fiscal (LFPRH)
Seguro Popular de Saúde 2006-2012 Consolidação	Governo Felipe Calderón Hinojosa − PAN (01/Dez/2006 - 30/Nov/2012) - Reforma fiscal para aumentar arrecadação - Tentativa de reforma com flexibilização das leis trabalhistas - Manutenção dos contratos privados na Pemex - Crise econômica internacional (2008-2009) Financiamento da saúde: Em 2006: Gasto total saúde % do PIB: 5,4% Gasto público saúde % do PIB: 2,3% Gasto privado % do gasto total saúde: 57,2% Gasto desembolso direto % gasto total saúde: 53,8%	José Ángel Villalobos − Ministro da Saúde (01/Dez/2006 - 09/Set/2011) - Deputado federal e presidente da Comissão de Saúde na Câmara dos Deputados (2000-2003) - Filiado ao PAN - Defesa da eficiência administrativa Salomão Woldenberg − Ministro da Saúde (09/Set/2011 - 30/Nov/2012) - Coordenou o Seguro Popular de Saúde (2009-2011)	Continuidade das reformas anteriores, incentivos à participação de serviços privados 2007: Programa Institucional do IMSS 2007-2012 com o objetivo de modernização e separação de funções 2008: Criação da Comissão Coordenadora de Negociação de Preços de Medicamentos e Outros Insumos de Saúde 2007: Plano da América do Norte para a Gripe Aviária e Pandêmica (NAPAPI) 2009-2010: Resposta à emergência sanitária do H1N1 2012: Promulgação da Lei da Associação Público-Privada (APP) - impulsionou as contratações privadas para oferta de serviços
Seguro Popular de Saúde 2012-2018 Declínio	Enrique Peña Nieto − Partido Revolucionário Institucional (PRI, 01/Dez/2012 - 30/Nov/2018) - Reforma energética e fiscal permitindo a entrada de capital privado no PEMEX, imposto sobre bebidas açucaradas - Conclusão da reforma trabalhista iniciada no governo anterior - Altos índices de violência - Relatos de corrupção Financiamento da saúde: Em 2012: Gasto total saúde % do PIB: 5,4% Gasto público saúde % do PIB: 2,9% Gasto privado % do gasto total saúde: 46,9% Gasto Desembolso Direto % Gasto Total Saúde: 40,6%	Mercedes Juan López - Ministra da Saúde (01/Dez/2012 - 08/Fev/2016) - Primeira mulher no cargo - Ligação com o PRI - Ocupou cargo de gestão na Funsalud José Narro Robles − Ministro da Saúde (08/Fev/2016 - 30/Nov/2018) - Ligação com o PRI	Continuidade do modelo do Seguro Popular de Saúde, ênfase no alcance da cobertura universal em saúde e no modelo de atenção 2012: Comissão de Proteção Social em Saúde (CPSS) anuncia alcance da Cobertura Universal de Saúde 2015: Modelo de Atenção Integral à Saúde (MAIS)
INSABI 2019-2022	Andrés Manuel López Obrador − MORENA (01/Dez/2018 - 30/Set/2024) - Conjunto de reformas denominado Quarta transformação - Reformas de aumento da regulação do Estado nos setores energético e PEMEX, retornando o *status* de empresas públicas - Ampliação dos programas com pensões destinados a idosos e populações vulneráveis - Criação de novas universidades no país - Reconhecimento tardio da importância da pandemia de COVID-19 - Alta popularidade nacional Financiamento da saúde: Em 2019: Gasto Total Saúde % do PIB: 5,3% Gasto Público Saúde % do PIB: 2,6% Gasto Privado % do Gasto Total Saúde: 50,8% Gasto Desembolso Direto % Gasto Total Saúde: 42,3%	Jorge Alcocer Varela − Ministro da Saúde (01/Dez/2018 − 30/Set/2024) - Forte influência do grupo da medicina social latino-americana liderado por Asa Cristina Laurell, professora da UAM-X. Dificuldades de articulação do novo Instituto com instituições e serviços existentes.	Extinção do Seguro Popular e criação de novo Instituto Nacional para universalizar cobertura 2018: Extinção do Seguro Popular de Saúde e criação do INSABI em prol de um Sistema Universal de Saúde 2019: Início da pandemia de COVID-19 com altas taxas de letalidade e excesso de mortalidade no país 2019: Mudanças na compra de medicamentos 2022: Criação das bases legais do IMSS-Bienestar e do Modelo de Atenção à Saúde para o Bienestar (MAS-Bienestar)
IMSS-Bienestar 2023-atual	Andrés Manuel López Obrador − MORENA (01/Dez/2018 − 30/Set/2024) Cláudia Sheinbaum − MORENA (01/Out/2024-atual) - Primeira presidente - Alta popularidade - Teve como promessa de campanha a manutenção das reformas iniciadas pelo seu antecessor, denominando as medidas como Segunda Parte da Quarta transformação Financiamento da saúde: Em 2022: (último ano disponível) Gasto total saúde % do PIB: 5,7% Gasto público saúde % do PIB: 3,0% Gasto privado % do gasto total saúde: 48,1% Gasto desembolso direto % gasto total saúde: 39,1%	Jorge Alcocer Varela − Ministro da Saúde (01/Dez/2018 − 30/Set/2024) David Kershenobich − Ministro da Saúde (01/Out/2024-atual) -Médico pela UNAM com experiências em gestão, atividades técnicas e científicas. - O grupo da medicina social vinculado à UAM-X mantém a influência nas reformas. - Adesão parcial dos estados, com resistências relativas à recentralização de funções e ao perfil político partidário.	Extinção do INSABI e busca de expansão de cobertura por meio do IMSS-Bienestar, com recentralização de funções 2023: Implementação do IMSS-BIENESTAR e recentralização do SNS 2023: Recentralização do financiamento por meio do Fundo de Contribuição para os Serviços de Saúde (FASSA, acrônimo em espamhol) e Fundo de Saúde para o Bem-Estar (Fonsabi, acrônimo em espanhol) 2023: Fim da pandemia de COVID-19 no México

INSABI: Instituto de Saúde para o Bem-Estar; PEMEX: Petróleos Mexicanos; PIB: produto interno bruto; UAM-X: Universidade Autônoma Metropolitana - Xochimilco; UNAM: Universidade Nacional Autônoma de México.

Fonte: elaboração própria com base nas diversas fontes da pesquisa. Dados de financiamento da saúde obtidos da Organização Mundial da Saúde (https://apps.who.int/nha/database/, acessado em Abr/2026).

Ainda que com a redução dos gastos por desembolso direto no total das despesas em saúde, de 52,2% em 2000 para 40,6% em 2012, observa-se a persistência de altos patamares desse gasto ao longo de todo o período. A proporção desse gasto aumenta para 42,3% em 2018 e cai para 39,1% em 2022 (Quadro 1), um valor ainda elevado, que sugere efeitos limitados das reformas na redução das despesas das famílias com saúde.

### Cobertura e utilização de serviços pela população: mudanças em um sistema segmentado

Para a melhor compreensão das repercussões das reformas em saúde no México, analisaram-se indicadores de cobertura e de utilização de serviços, bem como possíveis implicações das mudanças para a segmentação em saúde e para as desigualdades regionais.

A Figura 1 apresenta a proporção da população coberta por instituições de saúde, de 2000 a 2020. Os indicadores evidenciam redução da proporção de pessoas sem filiação institucional de 57% em 2000 para 26,2% em 2020. O IMSS permanece como a principal instituição do sistema, cobrindo mais de 30% da população em todos os anos analisados. Destaque-se o aumento da cobertura formal para a população sem seguridade. A combinação da população coberta pelo Seguro Popular de Saúde, INSABI ou IMSS-Bienestar saltou de 7,1%, em 2005, para 26,1% em 2020, um aumento de 268%. Não houve variações relevantes nos regimes específicos para servidores, nem na parcela da população vinculada às instituições privadas. Os dados sugerem que, apesar das fragilidades apontadas nas reformas implementadas, a cobertura do SNS mexicano expandiu.

**Figura 1:** Evolução da cobertura populacional percentual segundo instituições da saúde, México, 2000, 2005, 2010 e 2020.

IMSS: Instituto Mexicano de Seguridade Social; ISSSTE: Instituto de Segurança Social e Assistência Social dos Funcionários Públicos; MARINA: Secretaria de Marina; PEMEX: Petróleos Mexicanos. Fonte: Instituto Nacional de Estadística y Geografía ^34^. Nota: a soma das porcentagens pode ser maior que 100% devido à população que declarou estar afiliada a mais de uma instituição. Não estão computadas na figura as populações classificadas como “não especificado” e a afiliada ao Programa IMSS-Bienestar, voltado à população rural, pois só havia disponibilidade do dado para o último ano da série.

A cobertura por Entidades Federativas é apresentada na Tabela 1. Mesmo com a expansão do Seguro Popular de Saúde e depois do INSABI, estados como Hidalgo, Chiapas e Michoacán, apresentam um percentual de não cobertos superior a 30%. Outros estados, como Nuevo León, Coahuila e Sonora, registram maiores coberturas dos institutos da seguridade social.

**Tabela 1:** Cobertura populacional percentual por instituições de saúde, segundo estados. México, 2000, 2010 e 2020.

Entidade Federativa	IMSS (%)			ISSSTE, PEMEX, DEFENSA, MARINA (%)			Seguro Popular de Saúde e IMSS-Bienestar (%)			Não cobertos (%)		
	2000	2010	2020	2000	2010	2020	2000	2010	2020	2000	2010	2020
Aguascalientes	48,0	44,8	54,8	7,8	7,7	7,0	-	25,6	19,1	43,4	21,1	18,4
Baja California	44,7	43,7	52,9	5,2	7,2	6,7	-	15,0	13,7	38,1	28,8	22,2
Baja California Sur	42,4	41,9	51,2	16,9	14,8	14,4	-	18,3	17,5	39,2	22,8	16,2
Campeche	27,4	29,5	31,0	11,6	10,9	10,4	-	36,7	35,3	60,5	22,2	21,9
Coahuila de Zaragoza	61,8	56,4	66,3	7,1	7,0	6,6	-	10,8	5,8	28,4	21,9	19,0
Colima	37,7	40,1	49,5	8,3	8,3	8,4	-	32,8	24,6	48,2	17,3	16,8
Chiapas	11,7	13,0	12,1	5,1	5,3	5,4	-	37,3	45,8	77,8	41,7	32,7
Chihuahua	49,2	45,2	54,6	4,5	4,7	5,7	-	17,8	18,4	39,3	23,5	15,3
Ciudad de México/DF	36,6	34,3	42,1	14,4	13,6	13,5	-	10,6	13,1	45,9	33,6	27,2
Durango	38,3	35,0	40,6	10,8	10,5	11,0	-	22,0	22,1	49,2	29,8	25,2
Guanajuato	28,7	28,7	36,6	5,3	5,6	5,5	-	34,5	35,4	64,5	29,6	20,7
Guerrero	12,2	12,2	14,3	8,3	8,7	9,7	-	31,4	49,3	78,1	45,7	25,2
Hidalgo	21,7	19,7	24,1	7,6	7,8	8,6	-	37,1	35,6	69,6	33,8	30,1
Jalisco	41,2	41,4	49,7	3,1	3,1	3,1	-	17,0	14,5	53,8	34,5	29,4
Estado do México	31,2	29,5	35,2	5,7	7,7	7,8	-	17,5	20,3	54,8	40,4	33,4
Michoacán de Ocampo	20,6	21,7	25,4	5,7	6,1	6,5	-	25,0	28,9	72,0	44,4	37,6
Morelos	26,9	26,3	31,3	7,6	7,5	8,3	-	28,1	31,4	61,7	35,3	28,0
Nayarit	31,0	30,7	37,6	10,0	10,7	11,0	-	35,3	28,0	58,5	22,8	22,1
Nuevo León	59,6	56,6	62,2	4,1	4,1	4,3	-	10,7	9,7	31,2	20,3	18,5
Oaxaca	15,8	15,1	14,1	7,0	7,4	8,1	-	32,6	46,0	76,0	43,1	29,4
Puebla	20,1	19,8	22,0	4,0	5,2	5,3	-	22,8	41,3	71,4	49,3	29,2
Querétaro	40,8	39,7	47,3	4,8	4,5	4,4	-	27,7	24,8	52,6	25,2	20,5
Quintana Roo	38,0	39,1	44,5	8,3	7,5	6,6	-	19,2	20,7	51,5	29,8	25,2
San Luis Potosí	31,1	32,7	39,6	6,1	6,1	6,3	-	33,2	34,1	61,2	25,9	17,4
Sinaloa	45,3	41,5	50,1	8,2	8,6	9,3	-	24,4	20,8	45,4	24,5	19,0
Sonora	45,8	44,4	53,6	7,0	10,8	10,1	-	17,3	15,8	41,7	25,0	18,6
Tabasco	14,5	14,1	20,8	9,9	11,6	11,2	-	44,7	33,1	69,5	25,2	31,4
Tamaulipas	40,9	38,3	47,3	10,4	9,0	9,0	-	23,8	20,9	46,5	22,5	20,2
Tlaxcala	23,2	18,1	24,1	6,6	6,5	6,8	-	35,1	39,6	68,8	37,8	26,6
Veracruz de Ignacio de la Llave	23,7	24,8	28,1	7,2	7,3	7,4	-	25,3	35,1	68,1	39,9	27,6
Yucatán	39,3	38,9	42,2	5,9	5,6	6,1	-	28,8	27,1	53,9	24,1	21,8
Zacatecas	26,0	26,6	32,5	6,6	7,1	7,3	-	34,6	39,5	66,3	30,5	20,1
Total nacional	32,3	31,5	37,5	7,0	7,4	7,5	-	23,3	26,1	57,0	33,8	26,2

DEFENSA: Secretaria de Defesa Nacional; IMSS: Instituto Mexicano de Seguridade Social; ISSSTE: Instituto de Segurança Social e Assistência Social dos Funcionários Públicos; MARINA: Secretaria de Marina; PEMEX: Petróleos Mexicanos.

Fonte: Instituto Nacional de Estadística y Geografía ^34^.

Quanto à utilização de serviços, dados da ENSANUT sugerem continuidades e mudanças ao longo do tempo. A Figura 2 mostra que, em 2000 e 2012, cerca de somente 30% da população responderam que, quando necessitam de atenção à saúde, buscam serviços vinculados aos institutos corporativos da seguridade social. A proporção de pessoas que buscam os serviços públicos prestados pelas secretarias de saúde aumentou entre esses dois anos, período de expansão de Seguro Popular de Saúde de 35,3% em 2000 para 45,4% em 2012. A proporção de pessoas que responderam que procuram o setor privado diminuiu de 25,1% para 12,4% no mesmo período. Porém, é importante destacar que, em 2012, o inquérito passou a contabilizar a procura por consultórios privados adjacentes a farmácias (CAF, acrônimo em espanhol), que atingiu 8,4% entre os respondentes.

**Figura 2:** Evolução da utilização de serviços de saúde, por tipo de estabelecimento, segundo instituições de saúde, México, 2000, 2012, 2020 e 2023.

IMSS: Instituto Mexicano de Seguridade Social; ISSSTE: Instituto de Segurança Social e Assistência Social dos Funcionários Públicos; MARINA: Secretaria de Marina; PEMEX: Petróleos Mexicanos. Fonte: Instituto Nacional de Salud Pública ^35^. Utilizou-se a pergunta: “serviço/Instituição de saúde que você usualmente procura quando tem/teve alguma necessidade de saúde?”. Nota: não estão computadas na figura as populações classificadas como “não especificado” e “não sabe”; Programa IMSS-Bienestar refere-se ao programa voltado à população rural e o desenvolvimento integral da família ao Sistema Nacional para o Desenvolvimento Integral da Família (federal, estadual e municipal) que são serviços básicos também articulados ao IMSS; a Secretaria de Saúde corresponde à infraestrutura disponibilizada para o Seguro Popular e posterior IMSS-Bienestar instituição de saúde; a informação para “consultórios adjacentes a farmácias” não constava no inquérito de 2000; a informação para “não foi atendido” não constava nos inquéritos de 2020 e 2023.

No inquérito de 2020, é possível observar diferenças nessas proporções de busca por serviços de saúde, mas manutenção da alta procura por serviços privados (incluindo CAF e consultórios privados). A proporção da população que buscava serviços dos institutos da Seguridade apresentou discreta redução (de 30,6% para 26,8%) e a que referiu utilizar serviços públicos, maior redução (de 35,3% para 14,8%). Por outro lado, aumentou a procura por consultórios privados adjacentes a farmácias (de 8,4% para 17,2%) e do setor privado (de 25,1% para 36,3%), que juntos ultrapassaram 50%. Esse foi o primeiro ano da pandemia de COVID-19, em que a população pode ter evitado os serviços públicos de saúde e procurado alternativas de atendimento privado individual. Naquele momento, o sistema de saúde mexicano passava pela implantação do INSABI, o que também pode ter afetado as respostas.

Em 2023, após o momento crítico da emergência sanitária, observam-se novas alterações: aumenta a busca por serviços públicos dos institutos corporativos (somando 34,6%) e por serviços públicos das secretarias de saúde/IMSS-Bienestar, e cai a busca pelo setor privado. Porém, a procura por consultórios privados adjacentes a farmácias continua a aumentar (para 17,8%). Tais consultórios prestam atendimento ambulatorial, especialmente por médicos, pagos via desembolso direto dos usuários (média de 20 a 30 pesos), sendo anexos e conveniados a farmácias privadas, muitas vezes responsáveis por sua administração ^28^.

A utilização de serviços por entidade federativa, demonstrada na Tabela 2, revela imensa variedade territorial no que se refere à utilização de serviços públicos e privados, com diferentes combinações. Os estados em que o IMSS registrou a maior expansão de cobertura (Tabela 1) foram os que apresentaram maior crescimento na utilização de serviços desse instituto: Aguascalientes, de 38,8% para 70,8%; Colima, de 33% para 78,7% e San Luis Potosí, de 22,1% para 67,4%. Houve alta utilização dos serviços do Seguro Popular/IMSS-Bienestar nos estados de Guerrero, Hidalgo e Chiapas, que apresentam menor dinamismo econômico e menos recursos. Em alguns estados - como Coahuila - registrou-se alto uso de serviços do IMSS, com baixos percentuais de utilização de outros serviços públicos e alto uso de CAF (22,5%). Outros estados têm baixa utilização do IMSS e alta dos CAF, como Campeche (20,2%) e Michoacán (30,6%).

**Tabela 2:** Percentual de utilização de serviços de saúde por tipo de estabelecimento, segundo instituições de saúde, de acordo com os estados. México, 2000 e 2023.

Entidade Federativa	IMSS (%)		ISSSTE, PEMEX, DEFENSA, MARINA (%)		Secretaria de Saúde (Seguro Popular de Saúde e IMSS-Bienstar) (%)		IMSS-Bienestar programa e desenvolvimento integral da família (%)		Setor privado (%)		Consultórios adjacentes a farmácias (%)		Outra instituição (%)	
	2000	2023	2000	2023	2000	2023	2000	2023	2000	2023	2000	2023	2000	2023
Aguascalientes	38,8	70,8	5,1	0,0	27,0	9,2	0,2	1,5	24,4	3,1	-	15,4	1,6	0,0
Baja California	37,5	45,6	5,9	3,5	14,8	0,0	0,4	0,0	35,2	33,3	-	14,0	2,4	3,5
Baja California Sur	34,5	46,2	16,0	25,0	28,3	1,9	0,0	0,0	17,6	9,6	-	17,3	0,5	0,0
Campeche	17,7	39,3	7,3	7,9	48,6	25,8	5,8	0,0	15,7	5,6	-	20,2	2,1	0,0
Coahuila de Zaragoza	54,8	51,9	5,0	6,1	13,1	9,6	0,7	0,0	21,6	9,2	-	22,5	2,2	0,7
Colima	33,0	78,7	7,8	9,8	33,9	6,6	0,1	0,0	20,2	3,3	-	1,6	1,8	0,0
Chiapas	10,4	41,2	4,6	0,0	47,3	29,8	13,1	0,0	16,5	18,4	-	10,5	2,4	0,0
Chihuahua	39,8	47,7	4,2	7,0	19,0	23,7	0,4	2,0	30,7	9,3	-	8,2	3,5	2,2
Ciudad de México/DF	25,6	32,8	10,0	10,2	26,5	17,0	0,1	0,5	32,1	18,7	-	18,6	2,6	1,7
Durango	29,3	50,0	6,9	9,0	33,6	16,7	7,0	0,0	19,9	7,7	-	16,7	0,8	0,0
Guanajuato	22,2	25,7	3,6	4,0	25,7	30,5	0,0	0,0	42,7	16,7	-	22,0	1,4	0,9
Guerrero	9,8	3,0	4,4	8,0	59,8	65,0	0,1	0,0	22,2	19,0	-	5,0	0,4	0,0
Hidalgo	14,5	18,0	3,2	5,1	46,0	38,5	11,7	1,3	21,8	22,0	-	14,4	0,7	0,5
Jalisco	30,8	41,5	1,8	2,4	30,6	22,9	0,0	0,2	28,5	17,7	-	13,2	3,1	1,9
Estado do México	26,6	21,6	3,5	5,0	32,3	24,3	0,1	0,4	29,6	23,2	-	23,2	3,6	2,1
Michoacán de Ocampo	11,1	15,3	3,9	7,7	35,3	26,0	2,7	1,7	40,3	17,9	-	30,6	1,3	0,4
Morelos	21,1	21,7	7,2	4,9	38,5	27,5	0,1	0,1	29,2	22,1	-	21,2	0,8	1,8
Nayarit	23,4	35,7	6,6	4,8	44,6	25,6	2,6	5,3	18,3	16,0	-	11,2	1,4	1,3
Nuevo León	46,1	37,6	2,8	4,7	19,1	25,9	0,0	0,0	24,1	15,3	-	14,1	3,2	2,4
Oaxaca	6,4	7,6	6,0	12,4	57,2	52,4	15,6	0,0	10,6	21,9	-	2,9	0,9	2,9
Puebla	12,9	14,2	3,9	2,3	36,1	32,8	11,3	1,9	30,1	38,1	-	9,7	1,6	1,0
Querétaro	32,8	21,6	2,6	1,8	33,9	32,0	0,1	0,0	25,0	24,6	-	18,0	1,6	1,8
Quintana Roo	22,9	32,1	5,4	3,9	40,6	20,1	0,2	0,1	27,1	15,8	-	27,1	0,4	0,7
San Luis Potosí	22,1	67,4	3,6	2,2	35,5	10,1	6,4	1,1	26,7	6,7	-	12,4	1,4	0,0
Sinaloa	36,2	39,7	6,2	6,0	24,2	19,1	3,2	1,1	24,1	10,5	-	22,3	3,2	1,3
Sonora	31,7	42,9	8,6	6,5	29,2	20,1	0,1	1,3	23,8	13,4	-	13,7	2,8	1,7
Tabasco	9,2	11,7	6,5	17,2	58,1	37,4	0,1	0,0	17,5	12,6	-	18,0	3,3	3,0
Tamaulipas	30,6	34,2	8,1	0,0	24,1	13,7	1,1	19,2	29,2	5,5	-	27,4	3,2	0,0
Tlaxcala	19,3	20,2	4,4	5,3	42,4	37,8	0,2	1,2	29,5	23,4	-	10,5	1,6	1,6
Veracruz de Ignacio de la Llave	15,5	3,3	5,0	11,5	43,0	41,0	5,3	0,0	22,6	34,4	-	9,8	2,7	0,0
Yucatán	26,8	21,1	4,6	4,2	31,4	38,6	13,5	0,0	20,0	25,9	-	8,4	1,1	1,8
Zacatecas	19,0	3,9	4,4	13,2	40,7	34,2	4,6	0,0	27,2	40,8	-	7,9	0,6	0,0
Total nacional	26,25	29,29	5,48	5,35	35,32	27,23	3,44	0,71	23,96	18,43	-	17,84	1,85	0,88

DEFENSA: Secretaria de Defesa Nacional; DIF: desenvolvimento integral da família; IMSS: Instituto Mexicano de Seguridade Social; ISSSTE: Instituto de Segurança Social e Assistência Social dos Funcionários Públicos; MARINA: Secretaria de Marina; PEMEX: Petróleos Mexicanos.

Fonte: Instituto Nacional de Salud Pública ^35^.

Nota: não estão computadas na tabela as populações classificadas como “não especificado” e “não sabe”; Programa IMSS-Bienestar refere-se ao programa voltado à população rural e o desenvolvimento integral da família ao Sistema Nacional para o Desenvolvimento Integral da Família (federal, estadual e municipal) que são serviços básicos também articulados ao IMSS; a Secretaria de Saúde corresponde à infraestrutura disponibilizada para o Seguro Popular e posterior IMSS-Bienestar instituição de saúde; A informação para “consultórios adjacentes a farmácias não constava no inquérito de 2000; A informação para “não foi atendido” não constava nos inquéritos de 2020 e 2023.

## Discussão

O sistema de saúde mexicano, de base corporativa, passou por reformas nas últimas décadas que não romperam com a segmentação e fragmentação institucionais, historicamente relacionadas à vinculação ao trabalho e à heterogeneidade territorial. Isso se deve a assimetrias na cobertura das instituições de seguridade social - que, apesar do uso do termo “seguridade”, seguem a lógica de seguro social atrelado à inserção laboral − e na capacidade de oferta de serviços nos estados. Em uma região estruturalmente desigual, como a América Latina, tais características dificultam a redução de inequidades em saúde, que se acentuam em momentos de crise econômica com alto desemprego e informalidade laboral ^3^ ^,^ ^10^.

As reformas em saúde no período estudado, conduzidas por diferentes governos, enunciaram como objetivos a expansão da cobertura e a redução dos gastos privados em saúde, mas diferiram quanto aos princípios e às estratégias adotadas. Os processos reformistas foram influenciados por diferentes instituições e grupos de atores, bem como pela orientação política dos governos.

Algumas instituições públicas e privadas foram espaços de articulação de ideias e propostas, inclusive quanto ao sentido da atuação estatal e ao papel do setor privado. O Instituto Nacional de Saúde Pública, vinculado à Secretaria de Saúde, destacou-se na formação profissional, desenvolvimento de pesquisas e inquéritos, como a ENSANUT. A Funsalud, organização privada, formulou propostas de reforma do sistema de saúde e logrou que seus dirigentes ocupassem cargos no Ministério da Saúde ^4^ ^,^ ^10^. Ressalte-se ainda a atuação da Universidade Nacional Autônoma de México (UNAM) e da Universidade Autônoma Metropolitana − Xochimilco (UAM-X), no pensamento crítico, na formação de profissionais e de gestores atuantes no sistema de saúde ^3^ ^,^ ^10^.

Desde os anos 1980, dois grupos destacaram-se na proposição de agendas em saúde. Um deles é o grupo da “nova saúde pública”, sob a liderança de Julio Frenk, inicialmente vinculado ao Instituto Nacional de Saúde Pública, à Funsalud e à UNAM. O outro grupo, identificado com a medicina social latino-americana, teve como principal liderança Asa Cristina Laurell, professora da UAM-X, que atuou como secretária de saúde do Distrito Federal e dirigente no IMSS-Bienestar ^11^.

O primeiro grupo influenciou as reformas de 2000 a 2018, durante os governos de Vicente Fox, Felipe Calderón (ambos do PAN) e de Peña Nieto (PRI). A implantação do Seguro Popular de Saúde levou ao aumento das filiações e da cobertura de um conjunto limitado de intervenções, que se expandiu gradualmente. Porém, evidenciaram-se desigualdades entre estados no financiamento, gestão, filiação, qualidade e acesso aos serviços ^2^ e crescimento da contratação de serviços privados com recursos públicos ^3^ ^,^ ^20^. Em síntese, essa reforma resultou no aumento do asseguramento, mas reiterou a segmentação do sistema, limitou a expansão dos serviços públicos e favoreceu o setor privado por meio de incentivos ou de frágil regulação estatal. O desembolso direto em saúde permaneceu elevado ao longo do período, sinalizando um limite quanto a um dos propósitos enunciados da reforma: a proteção financeira.

Ainda que a reforma do México dos anos 2000 tenha diretrizes similares às do modelo do “pluralismo estruturado” da Colômbia - ênfase na expansão da cobertura, na separação de funções e na diversidade de prestadores públicos e privados ^5^ − há diferenças importantes. Na experiência mexicana não se rompeu o arranjo de cobertura dos trabalhadores formais por meio dos institutos corporativos, sendo adotado o Seguro Popular para expandir a cobertura dos pobres e dos trabalhadores informais a um pacote de serviços definido nacionalmente.

A partir de 2018, com a eleição de López Obrador, as propostas do grupo da medicina social ganharam espaço, destacando-se o fortalecimento do Estado nacional, recentralização de funções em saúde, extinção do Seguro Popular de Saúde, esforços de articulação de instituições e serviços públicos, e o compromisso com a contenção do setor privado. A criação do INSABI como estratégia para a expansão da cobertura e do acesso à população não segurada representou um esforço de universalização, mas esbarrou em limites estruturais, na fragilidade institucional, em conflitos federativos e entre grupos (dentro do governo e dos institutos), além do cenário da emergência sanitária.

O IMSS-Bienestar representou uma reorientação da estratégia do governo para expandir o acesso à saúde, que preconiza a recentralização da gestão, a articulação entre instituições e serviços existentes, e a orientação para um modelo com base na APS, apoiado em serviços públicos do IMSS-Bienestar, IMSS e ISSSTE. Porém, sua implantação, como as estratégias anteriores, depende da adesão dos estados (até o momento parcial) e dos institutos, dada a expectativa de articulação entre instituições e serviços.

No que concerne ao setor privado, ressalte-se o alarmante crescimento dos consultórios adjacentes a farmácias e de sua utilização pela população mexicana ^28^ ^,^ ^29^ ^,^ ^30^. Em 2021, havia cerca de 18.000 desses consultórios no país, com oferta média diária de 325 mil consultas médicas ^31^. O percentual de mexicanos que usualmente buscavam esse serviço mais do que duplicou entre 2012 e 2023, alcançando 17,8% ^30^, um dado preocupante, somado ao alto gasto por desembolso direto em saúde no país (39,1% em 2022, Quadro 1) ^32^.

A utilização dos CAF predomina entre pessoas sem seguridade social e com vínculos empregatícios precários, que buscam atendimento médico quando enfrentam problemas de saúde que as impedem de trabalhar. A procura se justifica pelo acesso rápido e baixo custo da consulta, que viabiliza o recebimento de uma prescrição médica e o retorno breve ao trabalho ^32^. Por se tratar de um serviço de saúde sem definição formal, junto a um estabelecimento comercial, tais consultórios se expandiram descolados do sistema nacional de saúde ^28^ ^,^ ^30^ e sob frágil regulação, carecendo de regras quanto ao seu funcionamento e fiscalização.

A procura por esses serviços cresceu durante a pandemia de COVID-19 devido à saturação dos serviços e ao risco de contágio nos hospitais ^3^ ^,^ ^30^ e se manteve elevada pós-pandemia, especialmente entre famílias urbanas de menor renda ^10^. Destacam-se como problemas a precarização do trabalho médico, a incerteza quanto à qualidade dos serviços ofertados, a falta de registros sobre os consultórios e seus atendimentos, e riscos de excessos na prescrição de medicamentos ^10^ ^,^ ^28^.

A proliferação desses consultórios dificulta o fortalecimento de um sistema público de saúde integrado baseado na APS, exacerba a mercantilização e a precarização da atenção. Tais consultórios oferecem serviços assistenciais básicos fragmentados e por demanda, que não substituem o cuidado que deveria ser assegurado na APS, segundo princípios da integralidade, territorialidade, continuidade e integração dos serviços. O Estado tem sido omisso ou frágil na regulação desses consultórios. Não há informações precisas sobre o número de consultas, de profissionais, medicamentos prescritos, queixas de saúde e localização dos estabelecimentos.

Nesse sentido, elementos do legado das reformas de saúde em décadas anteriores, como a persistente segmentação do sistema, o subfinanciamento público e a expansão de serviços privados, vão ao encontro do movimento de universalização do acesso à saúde enunciado pelas reformas recentes. A persistência de altos gastos por desembolso direto, associada ao aumento da utilização de consultórios privados, sugere limitações das reformas das últimas décadas em reforçar o sistema público, aumentar o acesso a serviços públicos e a proteção financeira em saúde.

No que se refere às limitações do estudo, o pequeno número de entrevistas não permitiu uma análise qualitativa das respostas, no que pese sua importância como fonte complementar para a compreensão do contexto das reformas, dado o conhecimento dos informantes-chave entrevistados. A opção por utilizar dados de duas fontes nacionais confiáveis (o censo e o inquérito ENSANUT) restringiu a análise a anos e indicadores disponíveis nessas fontes, utilizados como proxies da cobertura institucional e a utilização de serviços, com análise limitada das desigualdades no país. Há também limitações na análise dos dados de cobertura e acesso ao longo do tempo, por exemplo, pela não exigência de cadastramento das pessoas pelo INSABI ou IMSS-Bienestar. A expansão dos consultórios adjacentes a farmácias requer estudos específicos que explorem as mudanças na configuração público-privada em saúde no país. Por fim, o caráter recente e parcial da implementação do IMSS-Bienestar dificulta a análise de seus resultados.

Em 7 de abril de 2026, a presidente do México anunciou a criação de um Serviço Universal de Saúde, com credenciamento gradual de toda a população, que passará a ter direito de acessar todos os serviços de saúde ligados às diferentes instituições do país, independentemente do vínculo laboral ^33^. A implantação dessa reforma, a ser estudada nos próximos anos, pode representar um passo relevante para a universalização do acesso e a superação da lógica corporativa e segmentada que marcou historicamente o sistema de saúde mexicano.

## Conclusão

As reformas no sistema de saúde mexicano das últimas décadas, conduzidas sob governos de distintas orientações políticas, não superaram o legado histórico de segmentação por clientelas, fragmentação organizacional e desigualdades em saúde. Até o momento, o México mantém o acesso à saúde estratificado segundo a inserção laboral e a capacidade de pagamento, com diferentes status de cidadania na mesma sociedade. Não houve a criação de um sistema universal de saúde acessível a toda a população e nem mudanças estruturais que universalizassem o acesso aos serviços vinculados aos institutos da seguridade social.

As reformas no México foram influenciadas pela orientação dos governos e por disputas entre projetos de diferentes grupos. O Seguro Popular, apesar do aumento da cobertura formal de cesta restrita de serviços, reiterou a segmentação, aprofundou desigualdades, favoreceu a expansão de prestadores privados financiados com recursos públicos e não alcançou os objetivos previstos de cobertura universal e proteção financeira.

As reformas a partir de 2018, sob governos de centro-esquerda, buscaram a universalização do acesso com equidade, mas esbarraram em obstáculos estruturais, institucionais e políticos, o que levou à reorientação das estratégias. Observam-se movimentos de recentralização de responsabilidades e de recursos no governo federal, bem como a busca por maior articulação com as instituições corporativas e entre serviços de saúde.

Ao longo das sucessivas reformas, persistem desafios à universalização do acesso à saúde no México, como: insuficiente financiamento público; limitações de infraestrutura e de força de trabalho de saúde em muitos estados; resistências e riscos de não adesão dos estados e das instituições previdenciárias às propostas; conflitos federativos e político-partidários; expansão de serviços privados sob escassa regulação; e marcantes desigualdades territoriais e entre grupos sociais.

## Data Availability

As fontes de informação utilizadas no estudo estão indicadas no corpo do artigo.
